# Evaporation-Induced Polyelectrolyte Complexation: The Role of Base Volatility and Cosolvents

**DOI:** 10.1021/acs.langmuir.3c02656

**Published:** 2024-01-23

**Authors:** Jiaying Li, Martijn Hans Paul de Heer Kloots, Gerard van Ewijk, Derk Jan van Dijken, Wiebe M. de Vos, Jasper van der Gucht

**Affiliations:** † Membrane Science and Technology, MESA+ Institute for Nanotechnology, University of Twente, Faculty of Science and Technology, P.O. Box 217, 7500 AE Enschede, The Netherlands; ‡ Physical Chemistry and Soft Matter, Wageningen University and Research, 6708 WEWageningen, The Netherlands; § AkzoNobel, Decorative Coatings B.V., Rijksstraatweg 31, 2171 AJ Sassenheim, The Netherlands; ∥ BASF Nederland B.V., Innovatielaan 1, 8447 SN Heerenveen, The Netherlands

## Abstract

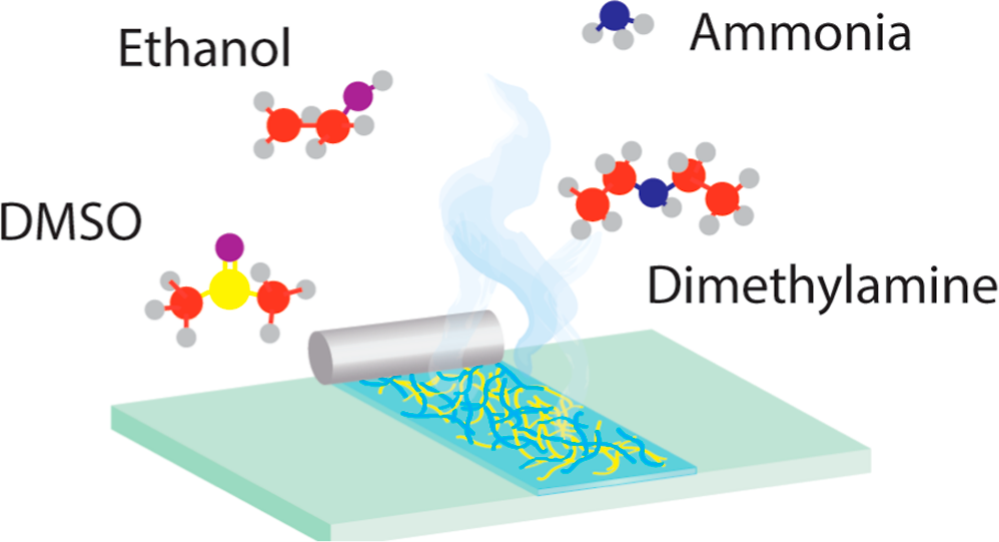

Film formation is a vital step for coating applications where a homogeneous, defect-free solid phase should be obtained, starting from a liquid casting formulation. Recently, an alternative waterborne-coating approach was proposed, based on the formation of a polyelectrolyte complex film. In this approach, an evaporating base induces a pH change during drying that initiates the complexation of oppositely charged polyelectrolytes, followed by further densification. In previous studies, ammonia was used as the evaporative base, leading to relatively fast evaporation and resulting in films showing significant brittleness, which tended to crack at low relative humidity or larger thicknesses. We hypothesize that slower complexation and/or evaporation can reduce the problematic stress build-up in the prepared polyelectrolyte complex coatings. For this reason, we studied the changes in the film formation process when there are different bases and cosolvents. We found that reducing the evaporation rate by changing ammonia to the slower evaporating dimethylamine or by adding DMSO as a cosolvent, led to less internal stress build-up during film formation, which could be beneficial for film application. Indeed, films prepared with ammonia showed cracking after 1 h, while films prepared with dimethylamine only showed cracking after one month. The fast evaporation of ammonia was also found to cause a temporary turbid phase, indicating phase separation, while for the slower evaporating bases, this did not occur. All prepared films remained sensitive to humidity, which poses the next challenge for these promising coatings.

## Introduction

Polyelectrolyte multilayers (PEMs) have gained great interest since their invention in 1992.^1^ Utilizing electrostatic interaction, sometimes complemented with other interactions, PEMs with controlled structures can be formed via repeated adsorption methods, including dipping, spraying, and spinning.^2^ The layer-by-layer (LbL) assembly of these PEMs can be controlled through the properties of the selected polyelectrolytes (type, charge density, and molecular weight) and the aqueous conditions, such as pH and salt concentration.^3,4^ With these straightforward but effective methods, versatile PEMs with specific functionalities have been prepared for use in many different research fields, for example, biomedicine, sensors, and separations.^5,6^

Although PEMs have shown great potential, their industrialization is limited by the extensive labor required for manufacturing. Therefore, researchers have been developing single-step methods by sedimentation^7^ or extrusion^8,9^ of polyelectrolyte complexes (PECs) and spin-coating^10^ or casting^11,12^ of polyelectrolyte coacervates. Recently, Pietsch et al.^13^ and our research group^14^ have showcased a single-step method by casting polyelectrolyte solutions. This unique method uses ammonia to bring polyethylenimine (PEI) to an uncharged state so that it can be homogeneously mixed with polyanions such as poly(acrylic acid) (PAA) or poly(4-styrenesulfonic acid) (PSS). After casting the homogeneous solution, the evaporation of ammonia and the resulting pH change lead to the positive charging of PEI, allowing complex formation with PAA or PSS. In our previous work,^14^ we have shown that this single-step method can be used to make dense polyelectrolyte complex films, which showed good gas barrier properties, making them interesting, for example, for applications in food packaging.

While these studies show the potential of this novel method, very little is known about the parameters that influence the film formation process and the quality of the final films. For example, when drying a polymer solution, internal stress develops as the solvent evaporates, which may lead to the formation of cracks.^15,16^ On top of the stress developed from concentrating the polyelectrolytes themselves, the strength and the degree of complexation also contribute to the mechanical properties. Indeed, we observed that PEI/PSS films, which have stronger ionic interactions than PEI/PAA, showed clear cracking behavior when their thickness exceeded a critical value.^17^ Unlike LbL assembly or the direct mixing of two charged polyelectrolytes, PEI gradually becomes charged as ammonia evaporates so that the complexation with PSS also proceeds gradually. Hence, the evaporation rate of the base determines the rate of complexation, which likely also influences the quality of the final films. This is similar to aqueous phase separation (APS), a method to create polyelectrolyte complex membranes, where coagulation baths were used to induce phase separation through control over the pH. Here, different pH values/buffer capacities determine the precipitation rate of PECs, resulting in different pore structures in the final membranes.^18,19^ Another example is vapor-induced polyelectrolyte complexation where chitosan was gradually charged by acetic acid vapor, after which it formed a complex with pectin.^20^ With this method, a more homogeneous and ordered network can be achieved, benefiting from the slow and steady evaporation of acetic acid vapor. In our case, we hypothesize that the complexation rate can be tuned by using bases with different evaporation rates.

Another approach to modifying the film formation process is to use cosolvents. For waterborne coatings, volatile organic compounds (VOCs) are added to aid film formation by lowering the *T*_g_.^21^ For PECs, water is a known powerful plasticizer.^8,22^ A posttreatment with high humidity can increase the mobility of chains and allow better PEC film formation.^11^ However, the use of organic cosolvents for PEC formation has rarely been studied. Cosolvents can shorten or prolong the drying time, depending on their volatility, which can influence the kinetics of the complexation process. Moreover, cosolvents will also change the dielectric constant of the solvent, which influences the dissociation of the polyelectrolytes and their conformations.^23−25^ We thus hypothesize that cosolvents may have a significant influence on the film formation process.

In situ characterizations are important to help us understand the film formation process. For the LbL approach, commonly used techniques to monitor the layer growth are quartz crystal microgravimetry (QCM), ellipsometry, and surface plasmon resonance (SPR).^5,26,27^ For PEC particles, light scattering, and optical observations are commonly used.^28−31^ One straightforward observation for PEC formation is their turbidity change.^31,32^ Solid-like PECs usually appear white in the wet state, while fluid-like complexes are often more transparent.^33,34^ When preparing APS-based polyelectrolyte complex membranes, the rate at which films turned white can even be used as a parameter to understand how fast the precipitation happens.^35^ In our evaporation-based method, a turbid intermediate phase was also observed. To capture this phase change, Laser Speckle Imaging (LSI) is a powerful tool that can be used. LSI has been developed to observe and quantify dynamic changes in soft matter, such as film drying or crack initiation.^36−38^ Here, it can help us to compare the film drying over time and with different bases on a microscopic scale.

**Scheme 1:**
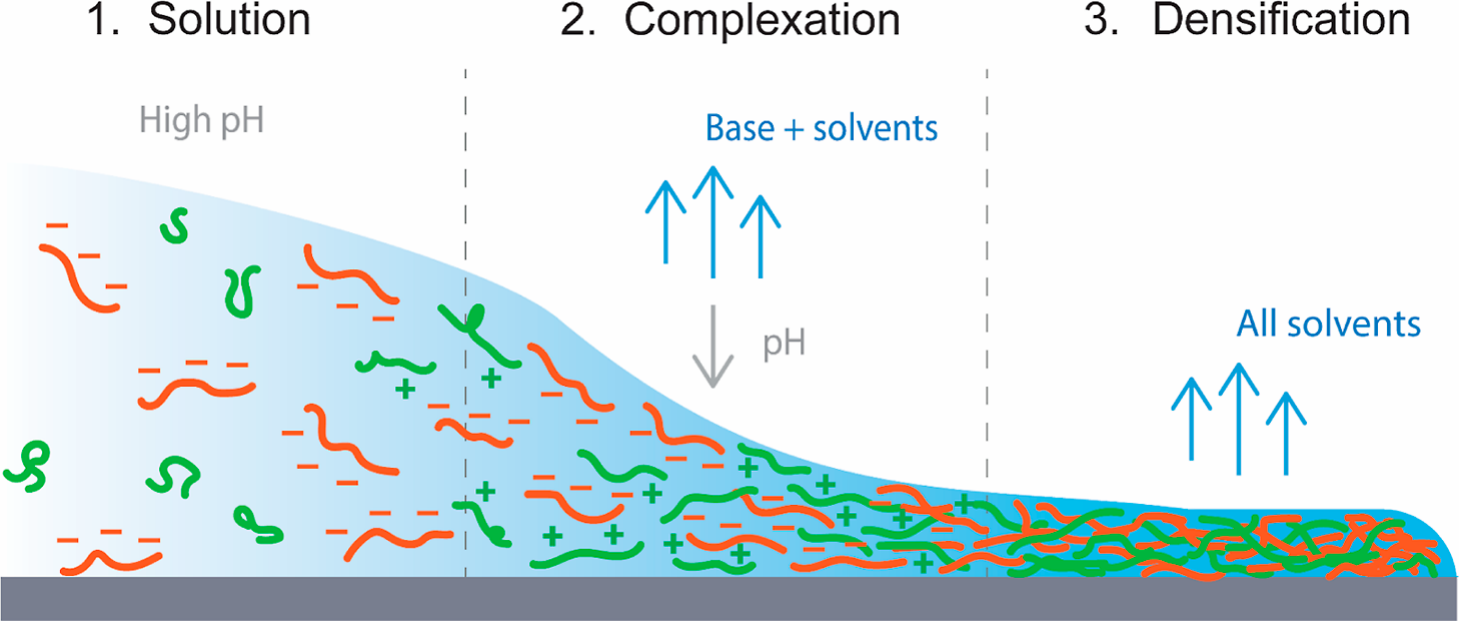
Film Formation Mechanism of Our PEC Coatings (PEI: Green, PSS: Orange); (1) A Homogeneous Solution is Obtained by Using a High pH at which PEI is Uncharged; (2) after Casting, the Evaporative Base and Solvents Gradually Evaporate Which Lowers the pH and Charges up PEI; (3) the Positively Charged PEI Forms Complexes with the Negatively Charged PSS, Eventually Resulting in an Ionically Crosslinked Polymer Film that Further Densifies as the Solvents Evaporate

In this work, we formulated PEI/PSS solutions with different bases, which directly influence the rate of complexation, and cosolvents, which tune the overall drying rate. Here, dimethylamine and sodium hydroxide were chosen to be compared to ammonia, where dimethylamine evaporates more slowly than ammonia, and NaOH does not evaporate at all. As cosolvents, we chose to add ethanol or dimethyl sulfoxide (DMSO) both of which have good miscibility with water. For hydrophilic PEI and PSS, the addition of ethanol not only decreases the solvent quality,^39,40^ but also increases the evaporation speed, thus worse film formation is expected. On the contrary, DMSO is polar and aprotic, and it has good solubility for hydrophilic PEs (e.g., PSS)^41,42^ and a much slower evaporation rate. To study the in situ drying of films and to link film formation to the final properties, we used LSI to monitor the dynamic changes during drying. Scheme 1 illustrates the different stages in the film formation process that we use. By keeping other drying conditions the same, we also compared the final film properties, such as mechanical properties and water uptake.

## Experimental Section

### Materials

Branched PEI (average *M*_w_ 25k g·mol^–1^, ≤1% water), poly(4-styrenesulfonic acid) (PSS, average *M*_w_ 75k g·mol^–1^, 18 wt % in water), ammonia (NH_3_, for analysis EMSURE ISO, Reagent. Ph Eur, 25% in water), dimethylamine solution [(CH_3_)_2_NH, 40 wt % in H_2_O], sodium hydroxide (NaOH, >98% pellets), ethanol (absolute, ≥99%), DMSO (ACS reagent, ≥99.9%), and thymol blue (ACS reagent) were purchased from Sigma-Aldrich (The Netherlands). A 64.9 wt % TiO_2_ particle suspension in water (average diameter ∼ 260 nm, determined by dynamic light scattering) was received from Akzo Nobel Coatings BV, Sassenheim, The Netherlands. All chemicals were used as received. All water used was deionized water (Milli-Q, Merck, The Netherlands). Acetate sheets (250 μm thick) were purchased from JEJE Produkt, The Netherlands.

### Preparation of Casting Solutions

PSS was dried at 70 °C for 2 h to obtain a solid, and it was then stored at 30 °C under vacuum. To compare different bases, the same molar ratio of SS monomer/base = 1:4 was used (*M*_SS_ = 184.23 g·mol^–1^, *M*_ammonia_ = 17.03 g·mol^–1^, *M*_dimethylamine_ = 45.08 g·mol^–1^, and *M*_NaOH_ = 40.00 g·mol^–1^). However, with the same molar ratio of NaOH, the pH became so high that the polymer was degraded, thus it was reduced to SS/NaOH = 1:0.8. Twenty-five wt % stock PSS-base solutions were prepared maintaining these ratios. PEI solution was prepared by diluting PEI with water. PEI/PSS solutions were prepared by mixing PEI- and PSS-base solutions at a ratio of 2:1 or 3:1, this ratio was following the charged monomer ratio (M_EI_ = 43.04 g·mol^–1^ and M_SS_ = 184.23 g·mol^–1^). The final PE concentration was 30 wt %. While the NH_3_ solution was added, an ice bath was used to reduce evaporation during weighing.

To study the effect of cosolvent, ethanol and DMSO were chosen, and 25 wt % PSS–NH_3_ solution was used. For preparing the final PEI/PSS solution, pure PEI was diluted by ethanol/DMSO instead of water. After the PEI was mixed homogeneously in ethanol/DMSO, the PSS–NH_3_ solution was gradually added. At a ratio of PEI/PSS 2:1, the final solution consisted of around 30 wt % PE, 53.8 wt % water, 7.6 wt % NH_3_, and 8.6 wt % ethanol/DMSO. At a ratio of PEI/PSS 3:1, the final solution consisted of around 30 wt % PE, 46.5 wt % water, 6.5 wt % NH_3_, and 17 wt % ethanol/DMSO. The given sample name, vapor pressure, and surface tension (liquid–air interface) of all used bases/cosolvents are listed in Table 1. Because a higher vapor pressure value indicates faster evaporation, the mixture with ethanol should evaporate faster, while the mixture with DMSO should evaporate slower. We note that the reported vapor pressures correspond to the values for the pure liquids and that the actual vapor pressures for the mixed solvents would be different. However, since we only use these values as a qualitative indicator of the volatility of the various components, we have not attempted to obtain more accurate estimates for the actual vapor pressures.

**Table 1:** PEI/PSS Samples Were Named According to the Bases or Cosolvents^a^

sample name	base	cosolvent	vapor pressure (Pa)	surface tension (mN·m^–^^1^)
PEI/PSS–NH_3_	NH_3_		NH_3_ 857.1k^45^	
PEI/PSS–dimethylamine	(CH_3_)_2_NH		(CH_3_)_2_NH 170.3k (Sigma-Aldrich)	∼26 (at 25 °C)^46^
PEI/PSS–NaOH	NaOH			
PEI/PSS–ethanol	NH_3_	ethanol	ethanol 5.87k^47^	∼23^48^
PEI/PSS–DMSO	NH_3_	DMSO	DMSO 56^49^	∼43^50^

a Their vapor pressure and surface tension values (at 20 °C) are summarized (for water: vapor pressure 2.34k Pa, surface tension ∼73 mN·m^–1^).^43,44^

For the LSI measurements, 1.54 wt % of TiO_2_ suspension (64.9 wt %, therefore 1 wt % TiO_2_ particles) was added as strongly scattering tracer particles, which substituted a part of the solvent to maintain 30 wt % polyelectrolyte concentration. The studied ratio was PEI/PSS 3:1. PSS was first dried in an oven at 70 °C, after which water and volatile base were added. After the PSS was redissolved, PEI was gradually added to the mixture. Finally, the TiO_2_ suspension was added dropwise. The resulting mixture was stirred overnight to ensure a good dispersion. Before usage, the samples were gently homogenized again and left stationary until the bubbles were gone from the mixture to not interfere in the LSI measurement.

### Film Fabrication

Films were cast by using a BYK automatic film applicator (USA). A casting bar with a gap height of 500 μm was used for all films. Free-standing films were prepared on acetate sheets and dried inside a fume hood at around 20 °C and the relative humidity (RH) was around 40%. The final thickness was measured by a micrometer. The average result of 10 random locations on the film with standard deviation is reported.

To observe the film formation process, videos (included in Supporting Information, X100 real time) were made by recording the films right after casting. All videos were taken under the same lighting. To trace the pH change, thymol blue was added (small amount ∼0.001 g in 9 g mixture) into the solutions. After casting, the color change caused by volatile base evaporation was recorded (videos included in the Supporting Information, X100, real time). Since PSS solution is yellow and shows a slight color difference at different pH values (Figure S1a), a pH-color chart was created by linking the color of 10 wt % PSS–thymol blue solution to different pH. A 10 wt % PSS solution has a pH around 1. Starting with a 20 wt % PSS stock solution, NaOH solution was gradually added to reach different pH values (3, 5, 7, 9, 11, 13). The pH values were detected by pH paper (pH 0–14 Universal indicator, Merck, The Netherlands). The final 10 wt % was maintained by adding water, and thymol blue was added (Figure S1b). The solutions were stirred for 30 min. Finally, the solutions were dropped on acetate sheet with a white paper as background and photographed. The colors were extracted from the digital photographs by an eye dropper tool (Figure S1c).

### Film Characterizations

#### LSI

LSI measurements were done on a home-built setup, similar to the one used in the work of van der Kooij et al.^37^ Temperature and RH were regulated within a climate box to be 22 ± 1 °C and 50% respectively. While drying the samples, the air in the climate box was refreshed at a rate of 10 L/min. During measurement, the residual weight of the coating was recorded, and the measurement was stopped when there was no longer a significant change in the mass over time. Instead of using a casting machine, the LSI samples were cast by hand by using a casting rod with a gap size of 500 μm. This was done to ensure that the LSI samples could be entered into the LSI chamber as quickly as possible after casting. Furthermore, to prevent any differences in scattering/absorption and defocusing due to curling of the substrates, the samples were coated on a glass slide.

In order to obtain data, the turbid sample was illuminated with a 532 nm expanded coherent laser (Cobolt Samba, 1000 mW), after which a camera recorded frames of backscattered light. Importantly, multiple scattered light was selected through a perpendicularly oriented polarizer, filtering out specular reflection and low-order scattering. From the speckle pattern in these frames, *g*_2_ intensity autocorrelation functions can be extracted over time via eq 1
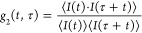
1Here, the obtained frames were spatially averaged over the field of view (640 × 480 pixels ≈2.9 × 2.2 mm). These averaged images were then used to compute the *g*_2_ autocorrelation functions, which quantify the fluctuations in intensity *I* over a timespan τ. After converting the *g*_2_ functions to the normalized field correlation functions *g*_1_ using the Siegert relation these can be fitted according to the following eq 2
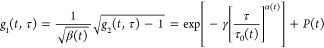
2where α(*t*) is the stretching exponent, β and γ are numerical constants and τ_0_ is the characteristic relaxation time of the system. Additionally, a plateau value *P* is added to account for incomplete decorrelation due to limitations of the measurement setup. Specifically, β gives a measure for the spatial coherence of the sample, while γ gives a measure for the distance that coherent light travels in the sample before it is converted to diffusive light. In the performed experiments and fit, β was obtained by measuring *g*_2_(t,0) directly from the measured frames, while γ was set to a value of 1.5.^37,51^ Thus, the fitted parameters are limited to *P*(*t*), α(*t*), and τ_0_(*t*). For more information on LSI and the processing of data, we refer to earlier studies by van der Kooij et al.^37^

#### Appearances

The appearance of the dry films was checked with an optical microscope (Leitz Ortholux, Germany). A scanning electron microscope (SEM, JSM-6010LA, JEOL, Japan) was also used. For this, the samples were first stored under vacuum overnight at 30 °C and then coated with 5-nm thick Pt/Pd (Quorum Q150T ES, Quorum Technologies, Ltd., UK).

#### FTIR and TGA

Fourier transform infrared spectroscopy (FTIR, Spectrum two, PerkinElmer, USA) and thermogravimetric analysis (TGA, NETZSCH, STA 449 F3 Jupiter, USA) were utilized to compare the chemical compositions of the dry films. For FTIR, the wavenumber range was from 400 to 4000 cm^–1^ under reflectance mode at a spectral resolution of 4 cm^–1^ (16 scans per measurement). The temperature range of TGA was from 40 to 800 °C with a ramp of 10 °C/min under a nitrogen environment. Once it reached 800 °C, it was held at this temperature for 5 min before cooling again.

#### Water Content and Water Uptake

The water content and water uptake of the freestanding samples were measured. The water content (%) was calculated based on eq 3
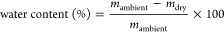
3where *m*_ambient_ is the weighed mass under ambient conditions (T 20 °C, RH 40–41%) and m_dry_ is the weighed mass after 4 h drying at 80 °C and stored in a vacuum oven at 30 °C overnight.

For water uptake, films were soaked in water for at least 24 h. Surface water was removed, and the wet weights (three sets) were measured. To obtain the dry weight, samples are dried in the oven at 80 °C and then stored under vacuum. The water uptake (%) was then calculated according to eq 4, with dry weight (*m*_dry_) and wet weight (*m*_wet_).
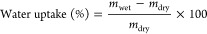
4

A leaching test was also conducted. The initial dry weight *m*_before_ was obtained by following the same drying procedures. Then, dry samples were soaked in water for 24 h, and they were rinsed thoroughly with running water. Lastly, the rinsed samples were dried again by following the same procedures to obtain *m*_after_. The leached wt % can be calculated as shown in eq 5
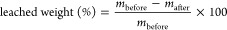
5

#### Tensile Measurements

Tensile measurements (Instron 5942, USA) were performed to compare the mechanical properties of different samples and how they changed over time. After drying under ambient conditions (24 h, 72 h, and 1 month), 5 cm × 0.5 cm (height × width) strips of samples were prepared. The thickness was measured by a micrometer at 4 random points. For each data point, at least three samples from different films were measured. The ambient conditions were RH 36–42% and T 20–21 °C.

## Results and Discussion

### Formation of Homogeneous Solutions

To make a homogeneous coating, it is important to start from a homogeneous coating solution. In our previous study,^17^ we found that sufficient NH_3_ should be added to a PEI/PSS mixture to avoid any complexation. The ratio between styrenesulfonate (SS) and ammonia that was used for making clear solutions was SS/NH_3_ = 1:5.73, but when the amount of NH_3_ was reduced to SS/NH_3_ = 1:3.14, small particles formed. In this work, different bases were compared. Since dimethylamine and NaOH both have higher molecular weight than NH_3_, the ratio SS/base was adjusted to 1:4 to be able to maintain a high PE concentration while still having a clear solution. However, at an SS/NaOH ratio of 1:4, the solution became so basic that the polymer degraded (Figure S2a). Therefore, the SS/NaOH ratio was adjusted to 1:0.8. All PSS-base solutions were basic, a 25 wt % PSS–NH_3_ (NH_3_ p*K*_a_ ∼ 9.3) solution had a pH value around 11, while PSS–dimethylamine (dimethylamine p*K*_a_ ∼ 10.7) and PSS–NaOH (NaOH p*K*_a_ ∼ 14) had higher pH values around 13 due to the higher p*K*_a_ values of the used bases (Figure S2b). All mixtures with different bases led to a homogeneous solution, and no complexation or phase separation was observed (Figure S2c).

For the mixtures with cosolvents, it was noticed that when adding PSS–NH_3_ into PEI–ethanol, a complex was formed which dissolved again later (Figure S 2d). A possible explanation for this is that locally the dielectric constant (ε_ethanol_ ∼ 25, ε_water_ ∼ 80) was so low that the electrostatic interaction increased.^39^ As more and more PSS–NH_3_ was gradually added, the dielectric constant of the mixture increased allowing the formed complexes to redissolve at high pH. This phenomenon was not observed for the sample with DMSO as cosolvent, likely because DMSO has higher dielectric constant (ε_DMSO_ ∼ 47). Moreover, for most organic solvents that are mixed with water, the dielectric constant of the mixture follows a linear relationship vs volume fraction, while for DMSO the dielectric constant decreases much more slowly.^52^ Using the described conditions, with all chosen bases and cosolvents, we were able to form homogeneous solutions that could be used for casting (Figure S2c).

### In Situ Studies

#### Turbid Phase during Drying

As discussed in the introduction, turbidity indicates that macroscopic phase separation is occurring. Here, we observed a large difference for films coated with different bases. When casting PEI/PSS films at a ratio of 3:1, PSS–NH_3_ films turned white, only to become transparent again at a much later stage in the drying process. On the other hand, for films cast with dimethylamine/NaOH as the base, no turbidity was observed (Figures 3a and S3b,c), indicating that phase separation is prevented by reducing the rate at which the pH decreases. Right after casting, films with dimethylamine did form a wavy pattern on the surface, which quickly disappeared (Figure S3b). The cause of this phenomenon is not yet clear, but it may be due to Marangoni flows caused by localized gradients in surface tension (Table 1) as a result of inhomogeneous evaporation, which can cause the elevation of certain parts of the film.^53^ Samples prepared with cosolvents both showed turbidity and thus phase separation (Figure S3d,e), possibly because the cosolvent reduced the solvent quality for the polymers compared to pure water. As a check, pure solvent mixtures (water and dimethylamine/ethanol/DMSO) were also prepared maintaining the same wt % ratio. As shown in Figure S4, no phase separation or dewetting was observed for all three combinations during drying on acetate sheets.

**Figure 1:**
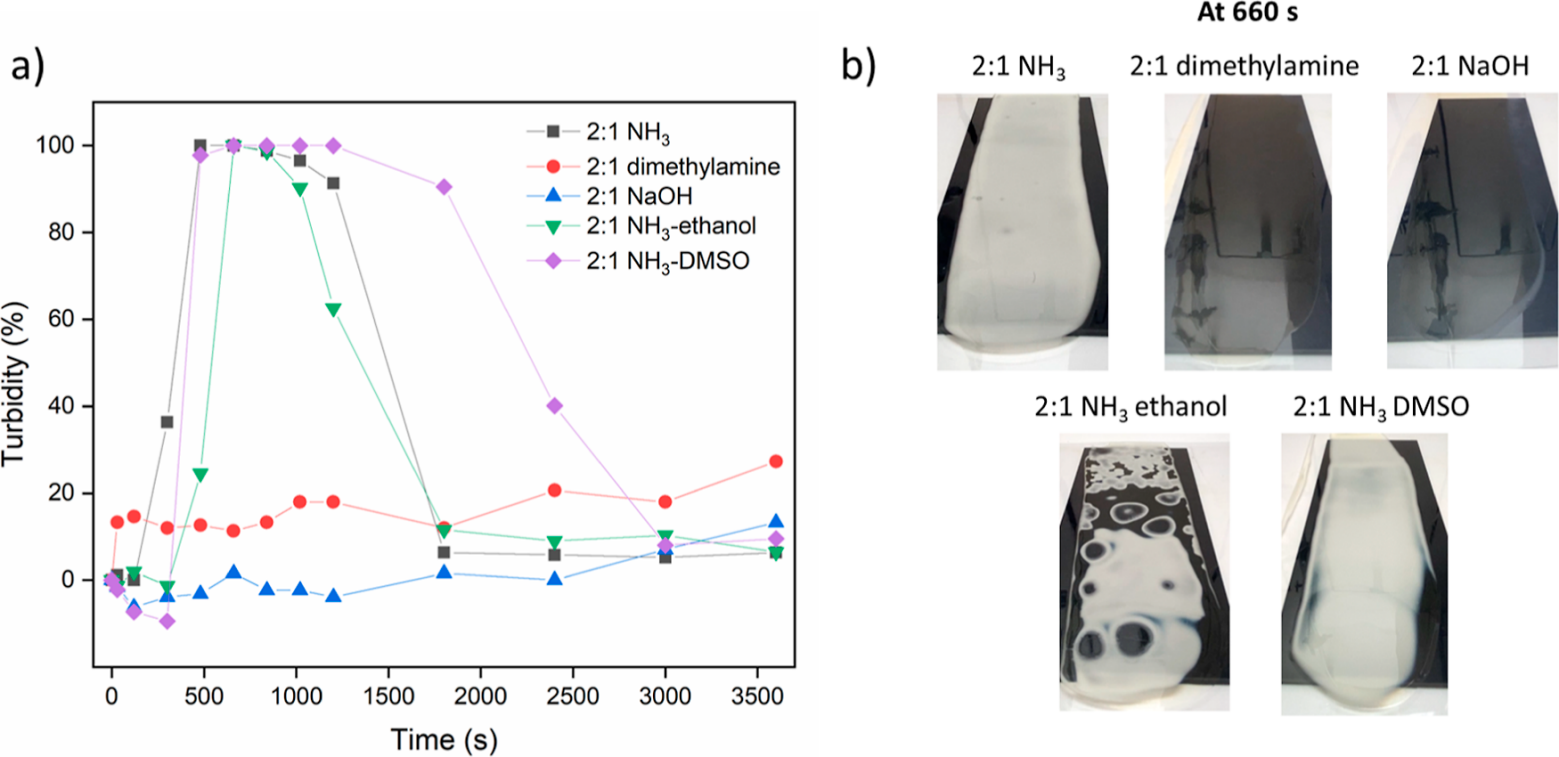
(a) Turbidity vs time of all samples at a PEI/PSS ratio of 2:1. (b) The appearance of films after drying for 660 s.

To further investigate the drying kinetics and especially the turbidity effects over time, videos of the various films at a PEI/PSS ratio of 2:1 were recorded during the first hour of drying. Snapshots of films with different samples drying vs time are shown in Figure S5. The turbidity (Figure 1a) can be extracted from the whiteness of the films as described in the Supporting Information (Figure S6 & eq S1). Films prepared with dimethylamine and NaOH remained transparent (Figure 1b) during the first hour of the drying process due to slow homogeneous evaporation or no evaporation at all of the base. However, for the NaOH film, crystallization gradually started from the edge at around 20 min, resulting in an opaque film after 1 h. By contrast, the PEI/PSS–NH_3_ sample also started to turn white from the edge and became fully turbid, while in the end the turbidity disappeared again. This phase was initiated at *t* ≈ 480 s and ended at *t* ≈ 1800 s. A possible reason is that once NH_3_ evaporates to a level where PEI starts to become charged, a PEC network is formed that holds the excess water in the pores. A further indication for this is that in this state, the film can be lifted as a whole, and when immersed in water, it remained as a complete film rather than dispersing (Figure S7). Unfortunately, this intermediate phase was too weak to prepare samples for electron microscopy. Even after soaking in 20 wt % glycerol or using liquid nitrogen to sustain the structure, it collapsed and became transparent upon drying. For the samples prepared with dimethylamine, the pH where PEC network formation starts was reached much later, when much more water has already evaporated, so that there is probably no need for pockets of water to be expelled. This may explain why these films remain transparent during drying.

As shown in Figure 1b, a turbid phase that indicates phase separation occurred for all samples with NH_3_, and the appearance of this phase separation depends on the cosolvent, suggesting that the nature of the separated phase and the phase separation process depend subtly on the solvent quality. The solvent quality of PEI/PSS–ethanol likely improves over time since ethanol evaporates earlier than water, while for PEI/PSS–DMSO, the solvent quality probably decreases since water evaporates faster than DMSO. Despite the phase separation, samples with NH_3_ all started to complex at around *t* ≈ 480 s since the base evaporation rate decides the initiation of complexation. Except for the DMSO sample, which could hold the turbid phase longer due to slower evaporation (til *t* ≈ 2400 s), all other films became transparent at *t* ≈ 1800 s.

#### pH Change during Drying

To better understand the drying process, the pH of the film was followed over time. This was achieved by adding a pH indicator, thymol blue, to PEI/PSS films at a ratio of 3:1 and subsequently monitoring the color change during drying. Typically, the color change of thymol blue vs pH started from blue (high pH) to yellow (around neutral pH) and then to red (acidic). In this case, we created an adjusted color vs pH chart since PSS itself is yellow (Figure S1c). The color changes of films versus time are summarized in Figure S8. By following the green color of the films (ratio between C and Y) and comparing that to the reference, we obtained an estimate of the pH of the films over time (Figures S9 and S10). Here, the values can only be used as an indication since the accuracy can be influenced by turbidity, lighting, and so on.

**Figure 2:**
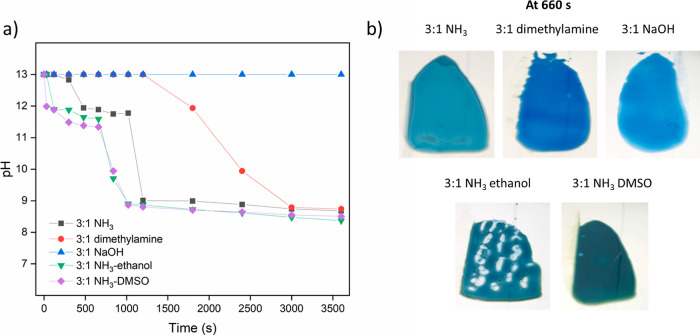
(a) pH vs time of all samples at a PEI/PSS ratio of 3:1. (b) The appearances of films with thymol blue after drying for 660 s.

As shown in Figure 2, the coating with NaOH as added base remained at high pH ∼ 13 since NaOH does not evaporate. For the PEI/PSS–NH_3_ sample, the pH dropped quickly during the first 1200 s. The pH change of the PEI/PSS–dimethylamine sample was more gradual since dimethylamine evaporates much more slowly. The addition of cosolvents did not have a large impact on pH and both PEI/PSS–ethanol and PEI/PSS–DMSO films showed a similar trend as PEI/PSS–NH_3_. The resulting films after 1 h drying all reached pH ∼ 9 where PEI should be partially charged. The pH values of these films did not change significantly after 1 h, although the loss of water may make further changes difficult to detect (Figure S9).

#### Turbidity Vs pH

**Figure 3:**
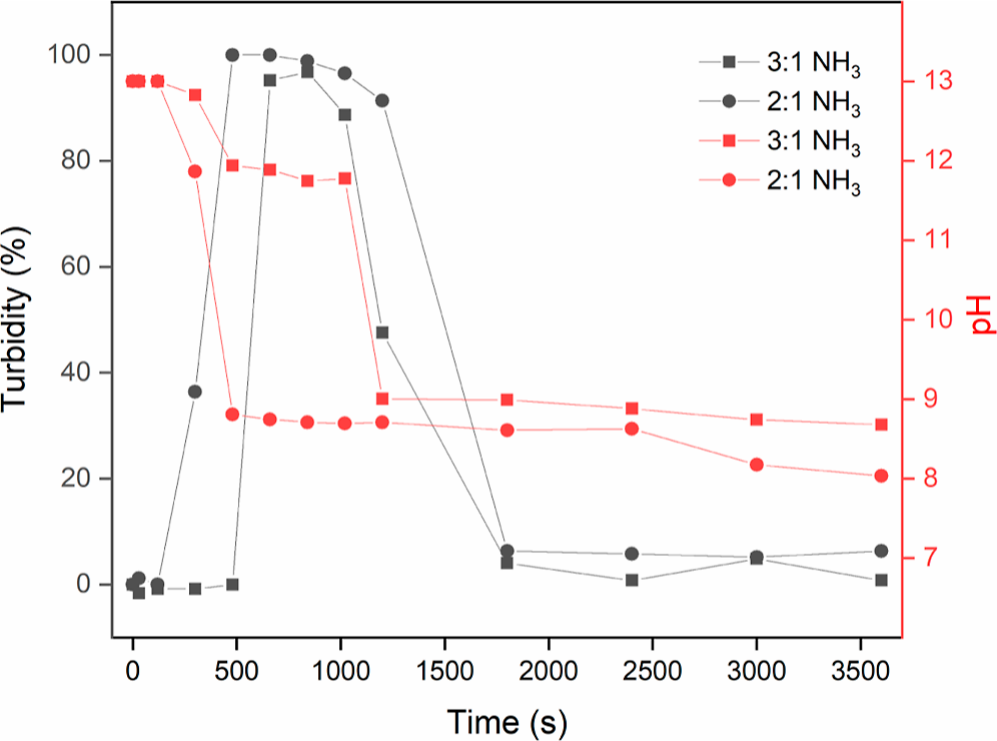
Turbidity and pH changes vs time for PEI/PSS–NH_3_ samples.

As discussed, the turbidity of the film is an indication that an inhomogeneous PEC network is formed. This phenomenon is directly influenced by pH since PEI becomes charged only at a sufficiently low pH, after which complexation starts. Indeed, Figure 3 shows that the onset of the turbidity increase occurred immediately after the pH has decreased to a value below ∼11.5–12. Branched PEI has 3 p*K*_a_ values 4.5, 6.7, and 11.6, corresponding to its primary, secondary, and tertiary amines.^19^ Hence, below 11.6, the PEI gradually becomes charged. When comparing PEI/PSS–NH_3_ at different ratios, the sample at a ratio of 2:1 showed a slightly stronger whiteness than the sample at a ratio of 3:1, probably due to a higher degree of complexation. PEI/PSS at a ratio of 2:1 can also reach lower pH since it contains less basic PEI than the 3:1 sample, which is also beneficial for complexation.

#### LSI on Samples with Different Bases

The dynamic processes during drying of the films were studied by using LSI, for which strongly scattering TiO_2_ particles were added as tracers. Here we only studied the samples PEI/PSS–NH_3_ and PEI/PSS–dimethylamine, since macroscopic phase separation (crystallization/domain formation) occurred in other cases (Figure 6). Any inhomogeneity in the LSI can affect the accuracy of measuring/averaging the relaxation times over a certain region of interest. LSI allows us to track the dynamics of a drying coating over time. More specifically, it determines changes in the local dynamics of scattering particles, which are then translated to the meso- or macroscale behavior of the studied material. The bitmaps recorded are used to derive *g*_2_ intensity autocorrelation functions (Figure S11), which are converted into *g*_1_ normalized field correlation functions (Figure S12), and subsequently fitted to obtain the characteristic relaxation time τ_0_. For more details on the LSI experiments, preprocessing of the data, and relevant equations, we refer to the Experimental Section.

**Figure 4:**
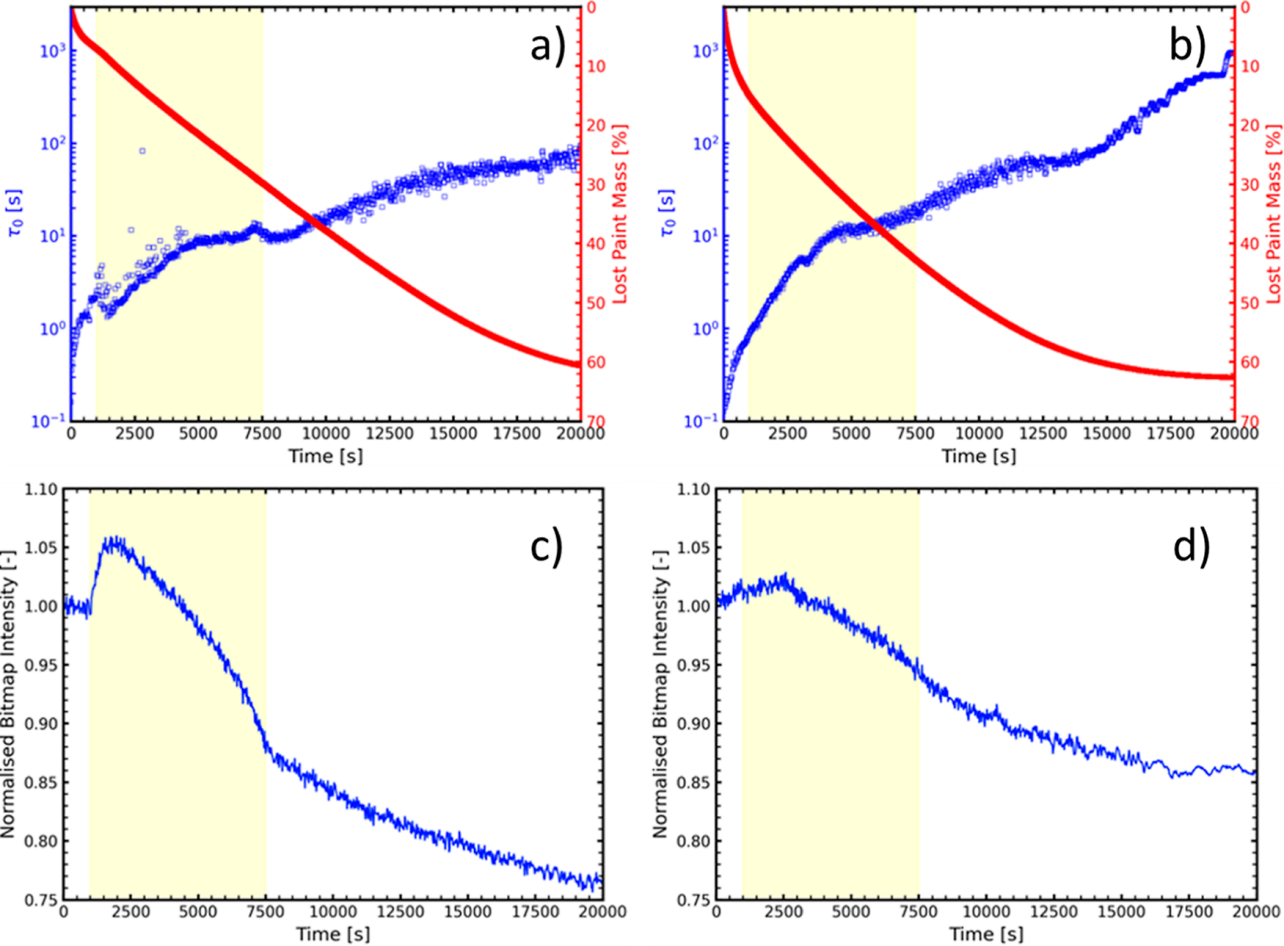
Results of LSI measurements for PEI/PSS 3:1 samples with NH_3_ (a,c) and dimethylamine (b,d) samples. The yellow region indicates the timespan of increased turbidity in NH_3_ samples (as seen in (c)). Top (a,b): Plots depict the characteristic relaxation time τ_0_ (blue) and the percentage of mass lost from the drying paint (red) over time. Bottom (c,d): The normalized bitmap intensity of the recorded bitmaps over time, as a measure of the scattering intensity.

The τ_0_ curves, as well as the changes in the mass of the films and a measure for the scattering intensity of samples with NH_3_ and dimethylamine are shown in Figure 4. τ_0_ is, by extension, a measure for the viscosity of the system, as it gives information on the diffusion coefficient of the TiO_2_ particles within the paint. Therefore, they can be used as a guide to determine the dynamics of the system over time. This is only valid if the TiO_2_ particles account for the complete scattering intensity, or at least represent the dominant contribution to the scattering intensity. In Figure 4a,b, we notice an overall quicker reduction in mass and a larger increase in τ_0_ for the system with dimethylamine, a somewhat counterintuitive result as NH_3_ would be expected to evaporate faster.

The likely cause of this result is that, since the molarity of base was fixed, there was more dimethylamine than NH_3_ in the films by weight (dimethylamine ∼17.3 wt %, NH_3_ ∼ 6.5 wt %). Although dimethylamine evaporates slower in terms of molecules per time unit, the higher molar mass can still cause a higher weight loss than for ammonia on initial time scales. Furthermore, the additional weight of dimethylamine in the sample replaces water, the slowest evaporating component, which determines the overall time before the coating reaches an equilibrium weight.

To test whether ammonia indeed evaporated faster than dimethylamine, the initial slopes of the weight loss were converted to an approximate molar amount of evaporated base per second per gram of applied coating. The values for this were 1.60 × 10^–5^ and 1.06 × 10^–5^ mol·s^–1^·g^–1^ for ammonia and dimethylamine, respectively, supporting the hypothesis that ammonia evaporates faster. The exact calculation can be found in the Supporting Information (Appendix 1).

From Figure 4c, the onset and presence of the turbid phase (*t* ≈ 1000 s) can be clearly seen in the scattering intensity of the NH_3_ sample. The presence of the turbid phase for the NH_3_ samples makes it difficult to assess the τ_0_ curve with confidence. As seen from the scattering data, the turbidity initially increases by roughly 6%, and it only seems to fully dissipate after *t* ≈ 7500 s. The later onset of the turbid phase (at about 1000 s instead of 660 s in Figure 1) can be explained by the higher RH (50%) and lower air flow (10 L/min) that was present in the LSI climate box. This also might have an impact on how long the turbidity is present, next to LSI being more sensitive to changes in turbidity than visual observation.

For polyelectrolyte systems similar to this, a quick pH change is known to lead to formation of a network structure, resulting in robust membranes.^54^ These membranes are typically turbid due to the presence of voids in its structure. We expect that a similar process is at play during the drying of the NH_3_ samples. This confinement of polymer-poor droplets in a polymer-rich network structure might not only alter the environment of the TiO_2_ particles, yielding a difference in τ_0_, but also impact the evaporation of water and base from the system. The mass data show that NH_3_ samples lose mass to evaporation (from water and volatile base) more slowly than dimethylamine samples. We hypothesize that this also is one of the causes for the slower increase in τ_0_ observed in NH_3_, when compared to dimethylamine, over longer time scales (e.g., 7500–20,000 s). In future studies, we will further address this effect and the existence of this turbid phase on the drying behavior of the coatings.

**Figure 5:**
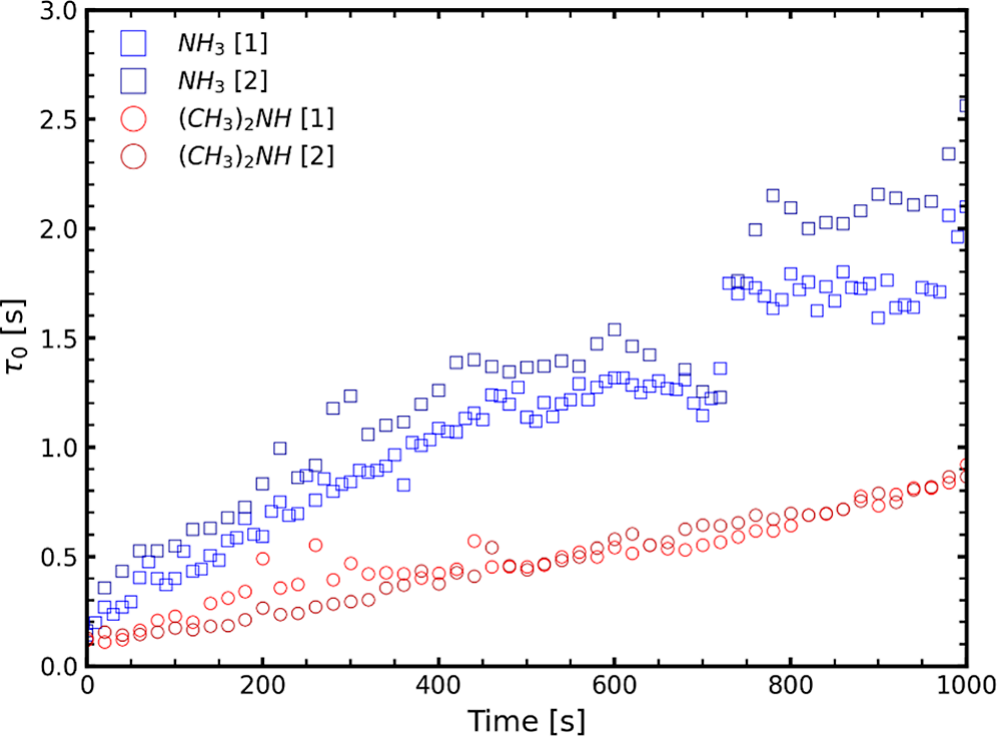
τ_0_ curves over time for NH_3_ and dimethylamine samples (in Duplo) during the initial drying stages.

Another more pronounced difference between the two bases can be seen in the initial stages of drying. When comparing τ_0_ for the NH_3_ and dimethylamine samples during the first 1000 s of drying, τ_0_ increases noticeably more rapidly for NH_3_ than for dimethylamine samples (Figure 5). This is expected since the faster evaporation of NH_3_ should lead to a quicker increase in charge density of the initially uncharged PEI, leading to polyelectrolyte complexation and an increase in viscosity of the solution. This also changes the mobility of the TiO_2_ particles and, hence, the characteristic relaxation time of the system. This initial increase in viscosity is of major importance for coating applications due to it being linked to the “open time” of the coating, during which reworking of the coating does not result in visible surface defects.

#### Physical Appearances of Dry Films

**Figure 6:**
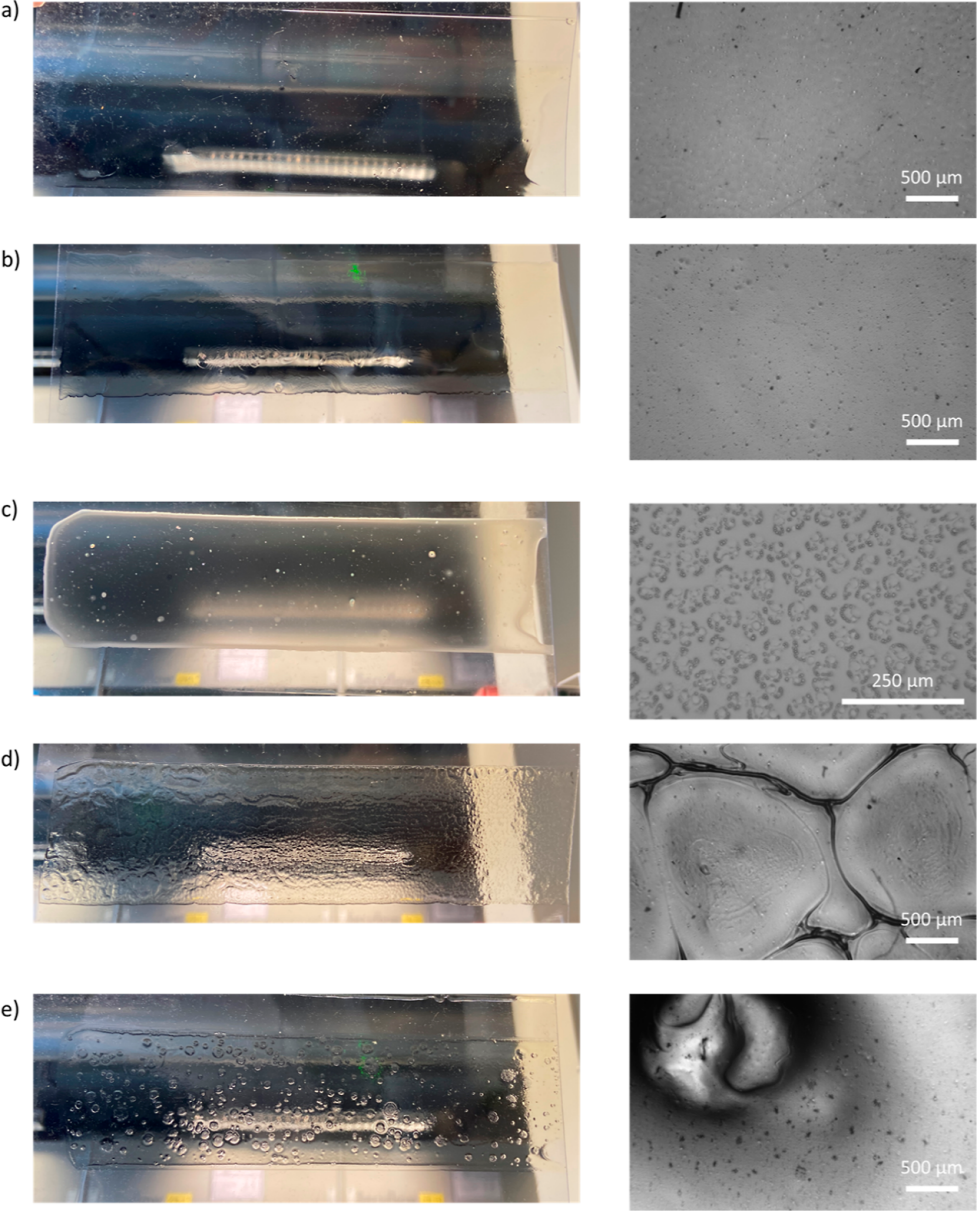
Physical appearance of dry PEI/PSS films with (a) PSS–NH_3_, (b) PSS–dimethylamine, (c) PSS–NaOH, (d) PSS–ethanol, and (e) PSS–DMSO at a ratio of 3:1. Images were taken 24 h after casting.

The appearances of the dry films were examined next. Figure 6 shows the appearance of PEI/PSS films at a ratio of 3:1. Films at a ratio of 2:1 showed similar morphologies, as shown in the Supporting Information (Figure S13). PEI/PSS–NH_3_ films appeared to be homogeneous (Figures 6a and S13a). PEI/PSS–dimethylamine films remained intact and also appeared homogeneous overall, but the wavy structure observed at the beginning of casting remained visible in the films (Figures 6b and S13b). For both ratios, PEI/PSS–NaOH samples showed crystallization, and the films were turbid (Figures 6c and S13c). Although the nonevaporative Na^+^ ions can shield SO_3_^–^, the effect on the final state is expected to be limited, especially due to them separating out of the films as crystalline domains. This crystallization behavior was consistent with our previous work where we observed crystallization of PEI/NaPSS films.^17^

Both PEI/PSS–NH_3_ and PEI/PSS–ethanol at a ratio of 2:1 cracked after drying for 1 h. The internal stress built up in these films slowly and gradually curled the substrates. Once cracked, the stress was released and films detached from the substrates (Figure S13a,d). This curling was not observed for PEI/PSS–dimethylamine and PEI/PSS–DMSO at a ratio of 2:1, indicating that slower evaporation of the base/solvent did help with reducing the stress. However, these films both cracked after much longer time scales of roughly 1 month (Table 2), probably because of changes in humidity, as it is well-known that PEC films become very brittle at low humidity. PEI/PSS–NaOH samples remained intact since there was no complexation, making it a much less brittle film.

**Table 2:** Cracking Behavior of Different PEI/PSS at a 2:1 Samples^a^

sample name	base	cracked after drying
PEI/PSS–NH_3_	NH_3_	1 h
PEI/PSS–dimethylamine	(CH_3_)_2_NH	1 month
PEI/PSS–NaOH	NaOH	
PEI/PSS–ethanol	NH_3_	1 h
PEI/PSS–DMSO	NH_3_	1 month

a All samples at a ratio of 3:1 remained intact, probably due to weaker complexation (Figure 6)

For all samples prepared with cosolvents, inhomogeneity was observed (Figures 6d,e, S13d,e). Samples at a ratio of 3:1 with cosolvents showed much more phase separation than those at a 2:1 ratio, probably because more cosolvent was added in these samples. Interestingly, spherulite-like crystals were observed for PEI/PSS–DMSO at a ratio of 3:1 (Figure S14). For polymers, this form of crystallization often happens with polymer thin films treated under certain kinetic conditions, such as supercooling of melts or evaporating concentrated solutions.^55−59^

After 1 month, SEM images were taken to check whether the films were dense (Figure S15). Indeed, all samples appeared to be dense except for the PEI/PSS–NaOH samples. More crystals had grown in the PEI/PSS–NaOH samples, which might generate defects and a porous film. In summary, changing NH_3_ to dimethylamine did not significantly change the film homogeneity, while the addition of cosolvents can lead to phase separation.

#### FTIR and TGA

FTIR was used to monitor chemical composition changes over time (24 vs 72 h). As shown in Figure S16a, there was no distinguishable difference among all PEI/PSS films at a ratio of 3:1. Dimethylamine residues could not be seen from IR since the peaks overlap with amine groups from PEI. Two small representative peaks (C–H bending from –CH_3_) from DMSO can be identified.^60,61^ After 72 h, the IR spectra of all samples were almost identical (Figure S16b). DMSO did evaporate more, as shown in Figure S16c.

**Figure 7:**
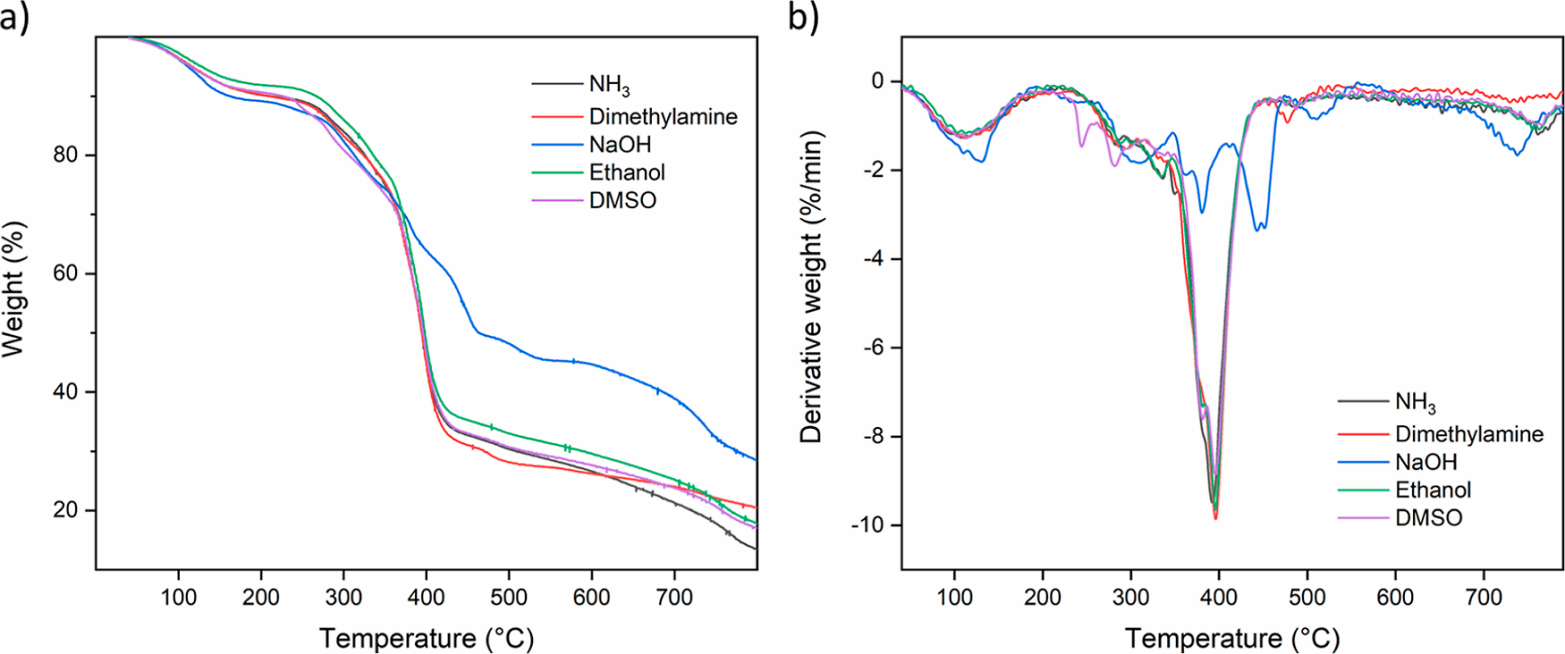
(a) TGA results and (b) derivative weight vs temperature of all films.

After 1 month, TGA measurements were performed to check the chemical composition of the final films. As expected, all samples prepared with an evaporative base show a similar decomposition trend, as shown in Figure 7. Samples prepared with NaOH had the most residue since NaOH all remained in the film. Also the pH remained high, and there was no complexation between PEI and PSS (Figure 7a). Instead of having one major decomposition peak around 400 °C, the PEI/PSS–NaOH sample decomposed as individual polyelectrolytes (Figure 7b). It can be concluded that the resulting films are chemically the same when all evaporative components are gone.

#### Water Content and Leaching

**Figure 8:**
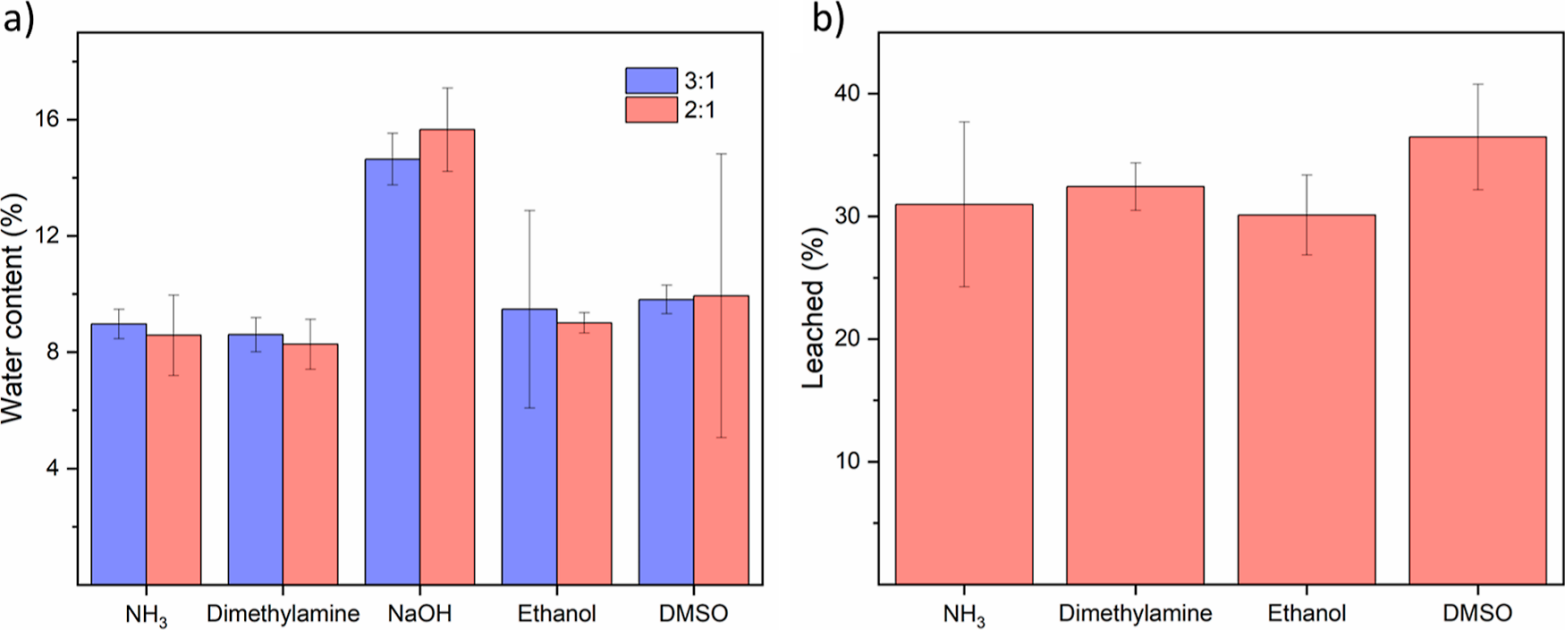
(a) Water content of PEI/PSS films at both ratios. (b) Leached % of PEI/PSS samples at a ratio of 2:1.

After 72 h, PEI/PSS films at ratios of 3:1 and 2:1 were soaked in water. PEI/PSS–NaOH samples dissolved since there was no complexation. Surprisingly, PEI/PSS–dimethylamine samples also dissolved (Figure S17a). When the films were removed from the substrates, dimethylamine could still be smelled, indicating that it was not fully evaporated, leaving the film at a higher pH so that PEI was not fully charged. After 1 month, PEI/PSS–dimethylamine samples swelled in water instead of dissolving, which indicates that dimethylamine did evaporate over time (Figure S17b). All PEI/PSS films at a ratio of 3:1 were partially soluble since this ratio has weaker complexation. The wet PECs were extremely soft and sticky, so the swelling/leaching test could not be done for this ratio. The water contents of PEI/PSS at ratios 3:1 and 2:1 after 1 month exposure to air are summarized in Figure 8a. All samples prepared with evaporative bases show similar values. Samples prepared with NaOH contained more water since there was no complexation and more free charges from PSS which can attract water. As shown in Figure 8b, the complexed films also showed similar leaching results. The swelling results of the remaining 2:1 films are shown in Figure S18. To sum up, there was no clear improvement in water sensitivity by changing the base or adding cosolvent.

#### Tensile Measurements

**Figure 9:**
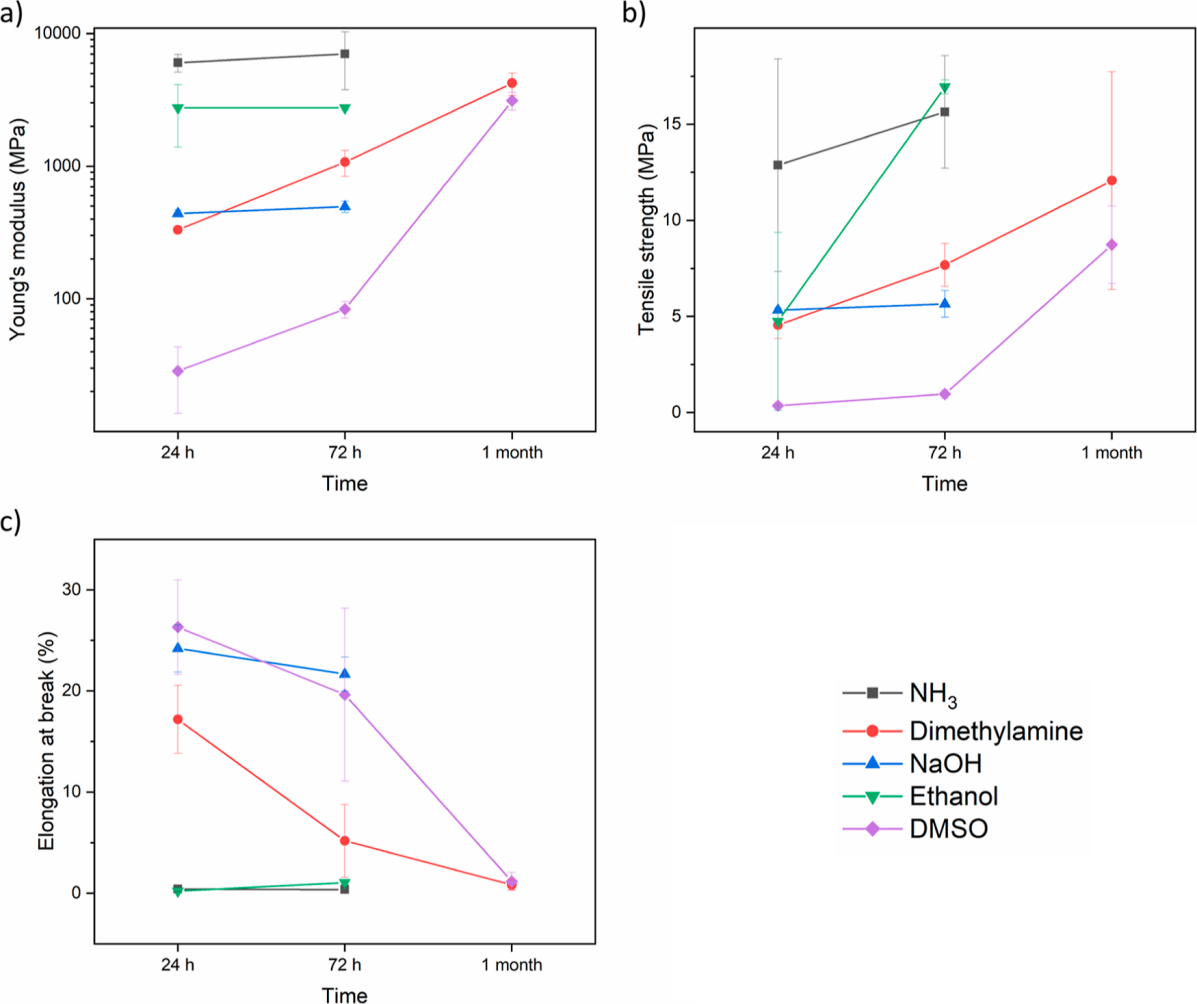
(a) Young’s modulus, (b) tensile strength, and (c) elongation at break of PEI/PSS at a ratio of 3:1.

To examine whether slower complexation/longer relaxation times can improve the mechanical properties, tensile measurements were conducted. The thicknesses of the films are summarized in Table S1. As shown in Figure 9, PEI/PSS–NH_3_ at a ratio of 3:1 was brittle with a high Young’s modulus and limited elongation. PSS–ethanol samples showed a similar brittleness. PEI/PSS–NaOH samples have much lower Young’s modulus and higher elongation at break since there was no complexation. For these three sets of samples, the mechanical properties were stabilized after 72 h since all solvents have evaporated, while PEI/PSS–dimethylamine and PEI/PSS–DMSO films slowly became more brittle over time. After 1 month, when almost all dimethylamine and DMSO evaporated, they showed no improvement in mechanical properties. Representative stress–strain curves are summarized in Figure S19.

PEI/PSS samples at a ratio of 2:1 showed a similar trend (Table S2). Eventually all films cracked after 1 month, except PEI/PSS–NaOH samples. Overall, samples at a ratio of 2:1 were more brittle than samples at a ratio of 3:1 since this charge ratio has a higher degree of complexation. To conclude, the clearly observable differences in dynamics leading to internal stress during drying were reduced in the final dry film, and the final mechanical properties after 1 month were quite similar. For conventional dispersion-based coatings, a longer open time allows the films to be repaired to avoid defects, which significantly improves the final film quality. After curing, the films are stable. However, PEC-based films often remain sensitive to changes in the environment, especially at low RH, where they become brittle.

## Conclusions

In summary, we compared different bases and cosolvents to better understand the film formation of PEI/PSS mixtures. With a nonevaporative base NaOH, there was no complexation but crystallization. With NH_3_, complexation happened fast, and a temporary phase separation was observed. Internal stress, built up in these films during drying, caused cracking of the films. With dimethylamine, a more homogeneous film formation was observed and the intermediate turbid phase and internal stress were absent. We anticipate that a more detailed understanding of the film formation process can be obtained by using microscopic observations, for example, using labeled polyelectrolytes, and by connecting these observations to the underlying phase diagram of the polymers. LSI was used to monitor the changes in relaxation time of NH_3_/dimethylamine samples, where subtle differences were observed. The reduced stress in the sample meant that the films were stable for a month before cracking occurred. However, this did not improve the mechanical properties and water sensitivity of the final films. The addition of cosolvents induced macroscopic phase separation during drying. The addition of DMSO did reduce cracking of the film, but once the DMSO had fully evaporated from the film, the film became brittle again. These findings show that tuning the rate of evaporation and complexation significantly alter the dynamics of film formation; however, the mechanical properties of the final films remain very sensitive to humidity and become brittle at low humidity. Compared to the LbL approach, preparing films in one step saves significant processing time. Decreasing the sensitivity toward humidity remains one of the challenges for these films.

## Supporting Information Available

Videos of film formation of different samples, color vs pH of PSS solutions, adjusting the pH of PSS solutions with different bases, observation of PEI/PSS at a ratio of 3:1 films drying, water–dimethylamine/ethanol/DMSO mixtures drying, capture of intermediate turbid phases, turbidity/color vs time of different films, color-pH conversion, LSI correlation functions, optical/SEM images of dry films, IR spectra of different films vs time, swelling tests, the thickness of different tensile samples, tensile results of PEI/PSS films at a ratio of 2:1, representative stress–strain curves of PEI/PSS films at a ratio of 3:1, and Appendix 1 which explains the weight loss of PEI/PSS–NH_3_ and PEI/PSS–dimethylamine LSI samples. The Supporting Information is available free of charge (PDF)
(ZIP)
