# Supplementary material for: Evaporation-Induced Polyelectrolyte Complexation: The Role of Base Volatility and Cosolvents
Source: Langmuir. 2024 Jan 23;40(5):2531–42. doi: 10.1021/acs.langmuir.3c02656 (PMC10851664; doi:10.1021/acs.langmuir.3c02656)
Supplement: Supplementary file 1 — la3c02656_si_001.pdf [file la3c02656_si_001.pdf]

SUPPORTING INFORMATION

Evaporation-induced Polyelectrolyte Complexation: the Role of Base Volatility and Cosolvents

Jiaying Li[1], Martijn Hans Paul de Heer Kloots[2], Gerard van Ewijk[3], Derk Jan van Dijken[4], Wiebe M. de Vos[1], and Jasper van der Gucht[2,]*

1. Membrane Science and Technology, MESA+ Institute for Nanotechnology, University of Twente, Faculty of Science and Technology, P.O. Box 217, 7500 AE Enschede, The Netherlands

2. Physical Chemistry and Soft Matter, Wageningen University and Research, 6708 WE Wageningen, The Netherlands

3. AkzoNobel, Decorative Coatings B.V., Rijksstraatweg 31, 2171 AJ Sassenheim, The Netherlands

4. BASF Nederland B.V., Innovatielaan 1, 8447 SN Heerenveen, The Netherlands

** Corresponding Author Jasper van der Gucht Email: jasper.vandergucht@wur.nl*

Number of pages: 22

Number of figures: 21

Number of tables: 2

Table of Contents

Figure S1. Color vs pH of PSS solutions---Page S3
Figure S2. Adjusting the pH of PSS solutions with different bases------------------------Page S4
Figure S3. Observation of PEI:PSS at a ratio of 3:1 films drying----------------------------Page S4
Figure S4. Water-dimethylamine/ethanol/DMSO mixtures drying-----------------------Page S5
Figure S5. Capture of intermediate turbid phases during drying--------------------------Page S6
Figure S6. Extracted turbidity vs time of different films-------------------------------------Page S7
Figure S7. The formation of a continuous film---Page S8
Figure S8. Capture of color changes during drying---Page S9
Figure S9. Extracted color vs time of different films---Page S10
Figure S10. Color to pH value conversion---Page S11
Figure S11. g_2 intensity autocorrelation functions (dimethylamine sample)----------Page S12
Figure S12. g_1 normalized field correlation functions (dimethylamine sample)------Page S12
Figure S13. Physical appearance of dry PEI/PSS at a ratio of 2:1------------------------Page S13
Figure S14. PSS-DMSO sample showed spherulite-like crystals--------------------------Page S14
Figure S15. SEM images of different samples---Page S15
Figure S16. IR spectra of different films vs time--Page S16
Figure S17. Swelling tests of different films---Page S16
Figure S18. Water uptake of PEI:PSS samples at a ratio of 2:1---------------------------Page S17
Table S1. The thickness of different tensile samples---------------------------------------Page S17
Table S2. Tensile results of PEI:PSS films at a ratio of 2:1---------------------------------Page S18
Figure S19. Representative stress-strain curves of PEI:PSS films at a ratio of 3:1----Page S19
Figure S20. Normalized mass decreases over time for ammonia and dimethylamine samples--Page S20
Figure S21. Lost paint mass and τ_0 over time during initial drying stages--------------Page S22

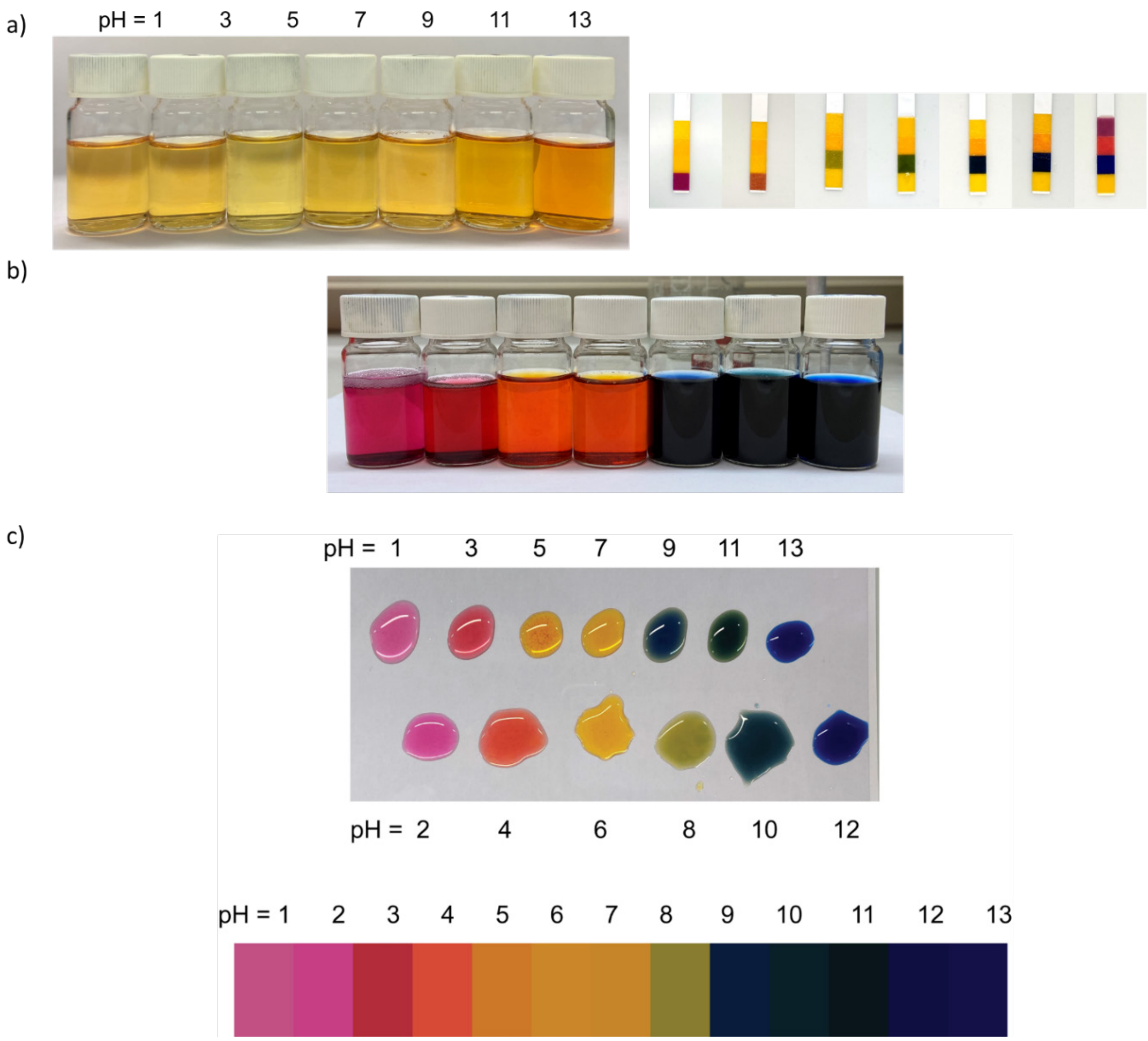

Figure S1. a) 10 wt% PSS solutions at different pH values. b) Thymol blue was added into the solutions to show different colors (~ 0.001 g thymol blue solid in 14 g 10 wt% PSS solution). c) The solutions were shown on an acetate sheet with a piece of white paper as background. By mixing same amount of 2 neighboring solutions (e.g. pH = 1 and 3), an indication color can be given for pH 2, 4, 6, 8, 10, and 12. Then, the colors were extracted with an eye dropper tool to build a color chart for different pH.

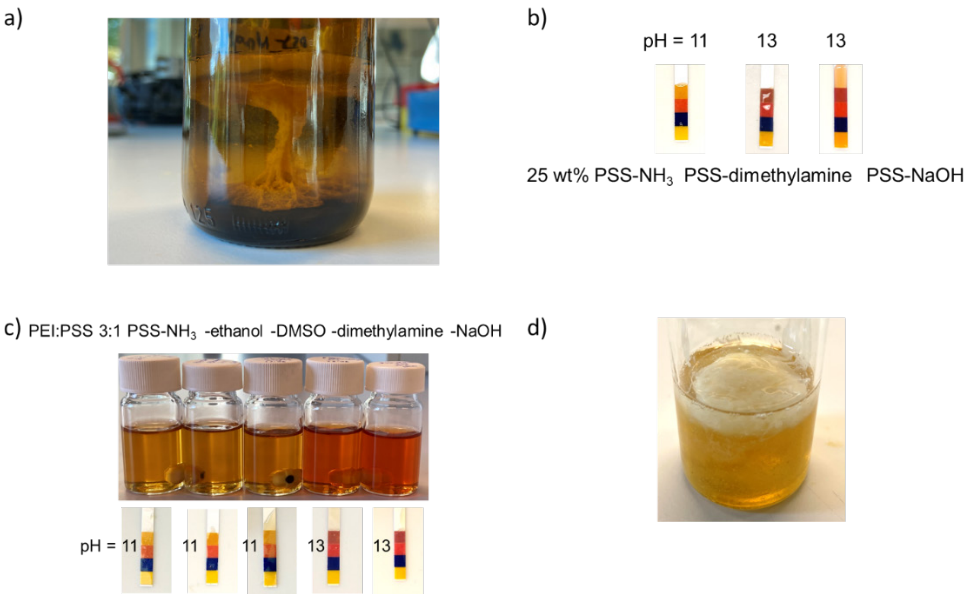

Figure S2. a) When keeping the SS:NaOH to 1:4, the basicity was so high that PSS was degraded. b) pH values of 25 wt% PSS solutions with different bases. c) Final mixture of PEI:PSS at a ratio of 3:1 with different bases and cosolvents. d) When adding PSS-NH_3 into PEI-ethanol, first complexation was observed, then it fully dissolved with the addition of PSS-NH_3.

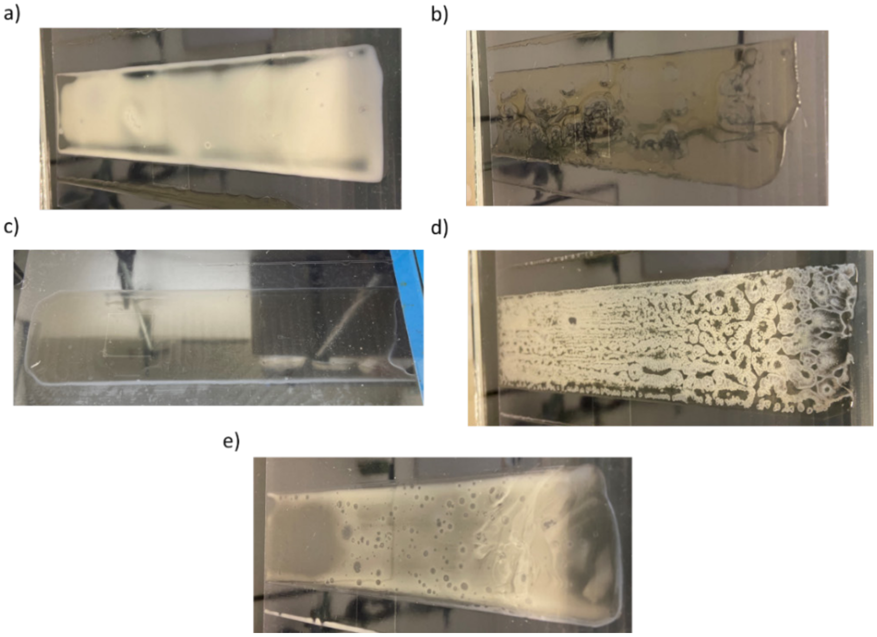

Figure S3. Observation of PEI:PSS films at a ratio of 3:1 with: a) PSS-NH_3, b) PSS-dimethylamine, c) PSS-NaOH, d) PSS-ethanol, and e) PSS-DMSO.

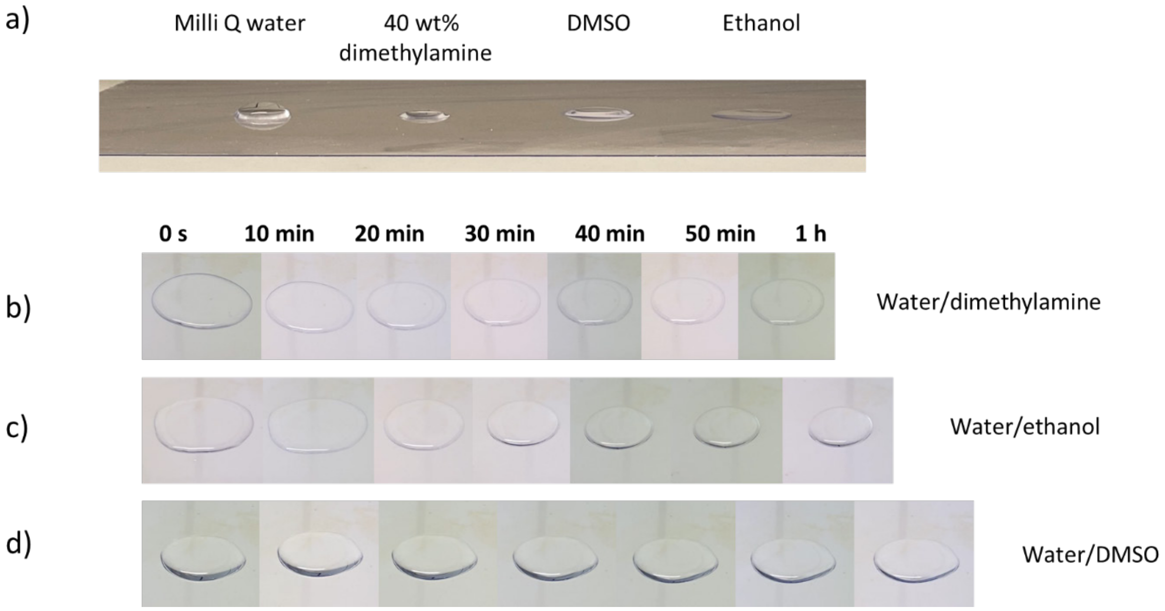

Figure S4. a) When dropping the same volume of solutions on acetate sheets, water remained a droplet, while ethanol spread out completely. DMSO and 40 wt% dimethylamine were in between. 5 mL of water and b) dimethylamine, c) ethanol, and d) DMSO mixtures were dried on acetate sheets (water/ethanol or DMSO 76/24 wt%, water/dimethylamine 75/25 wt%). During 1 h of drying, there was no dewetting or phase separation observed that they shrunk horizontally.

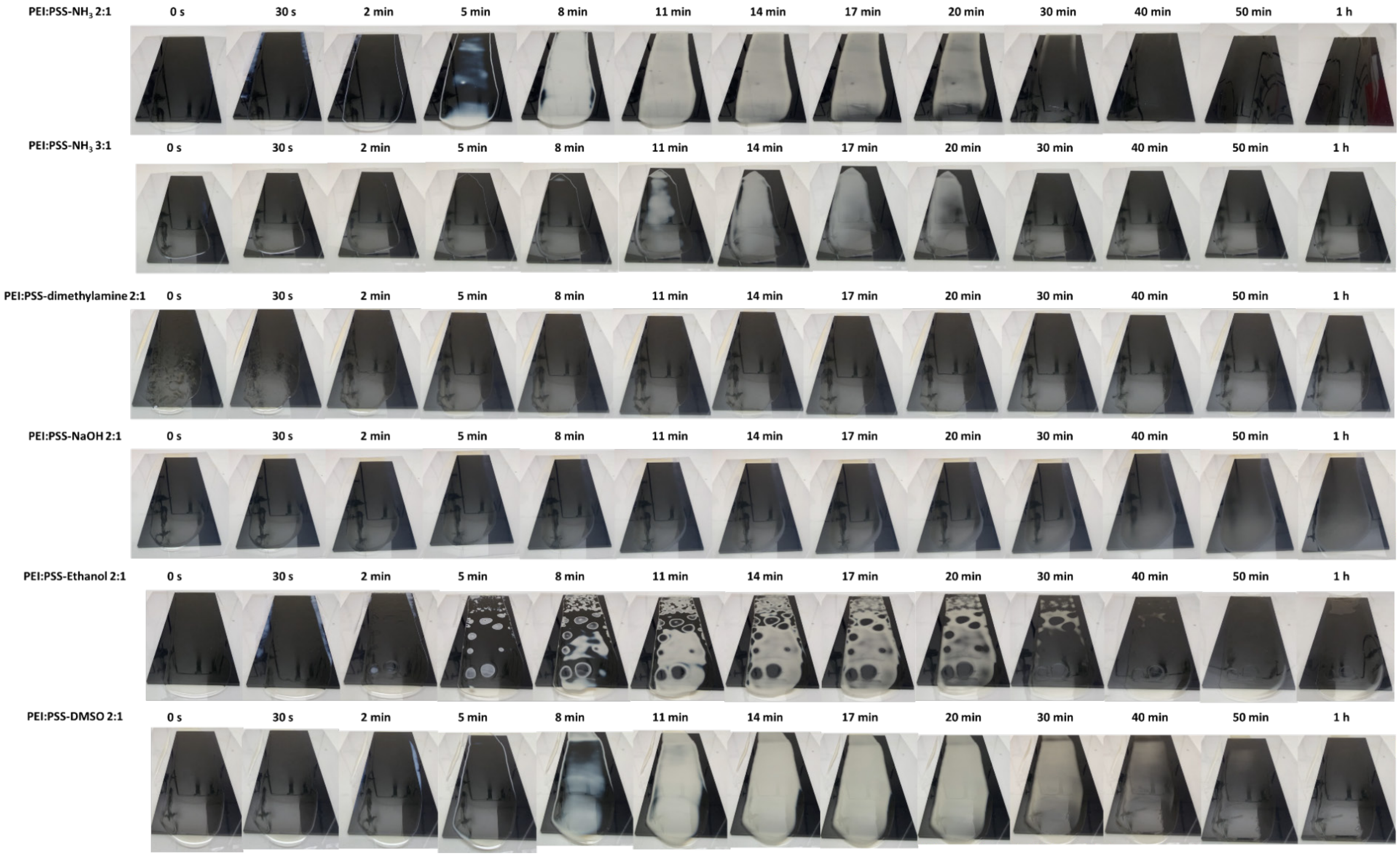

Figure S5. Screenshot pictures of different films drying vs time.

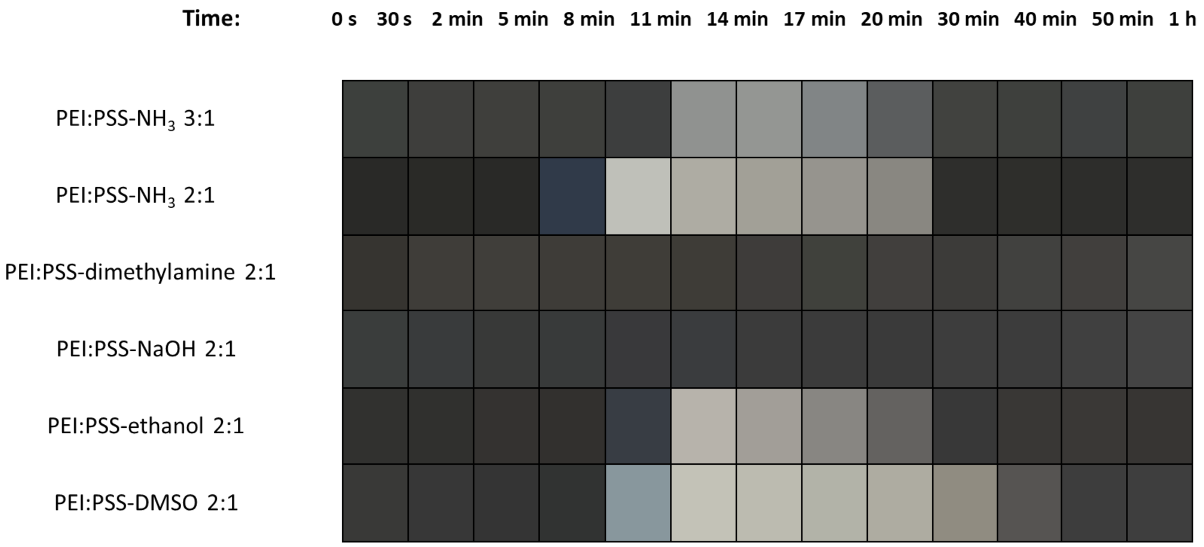

Figure S6. Turbidity change vs time of different samples. A black background was used and the colors were extracted using an eye dropper tool from a middle point, since the film drying was heterogeneous (e.g. edge dried faster, cosolvents phase separated). For samples with cosolvents, phase separation parts were avoided. The color of the same point was tracked vs time.

From the colors above, CMYK (cyan, magenta, yellow, key (black)) values can be obtained. Since we only need the turbidity (whiteness) information, we focused on the K value which determines how black the color is. K = 100 means black, while K = 0 means white. The turbidity of films can be calculated basing on Equation S1:

$$Turbidity\,(\%) = \frac{K_0 - K_t}{K_0} * 100 \qquad (S1)$$

where K_0 is the initial K value of the film at 0 s and K_t is the K value at a certain time t. When turbidity equals to 100%, it suggests the blackness of the substrate is fully covered.

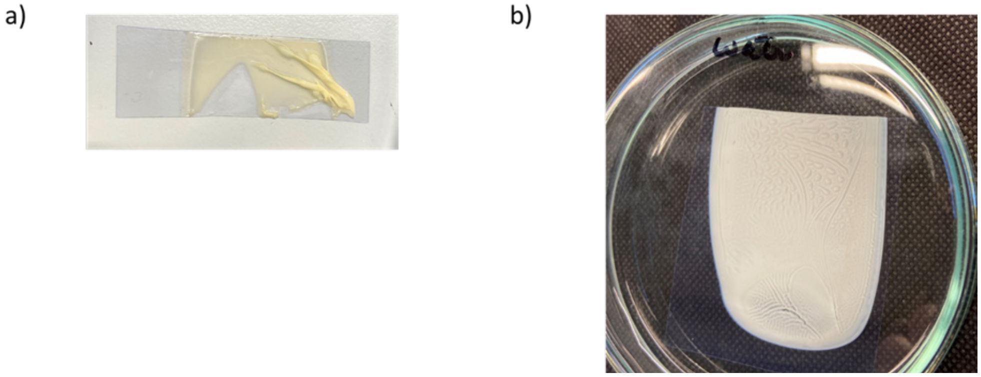

Figure S7. PEI:PSS-NH_3 at a ratio of 2:1 film turned turbid. a) At this stage, a continuous film was already formed. b) When immersed in water, the film remained as a whole.

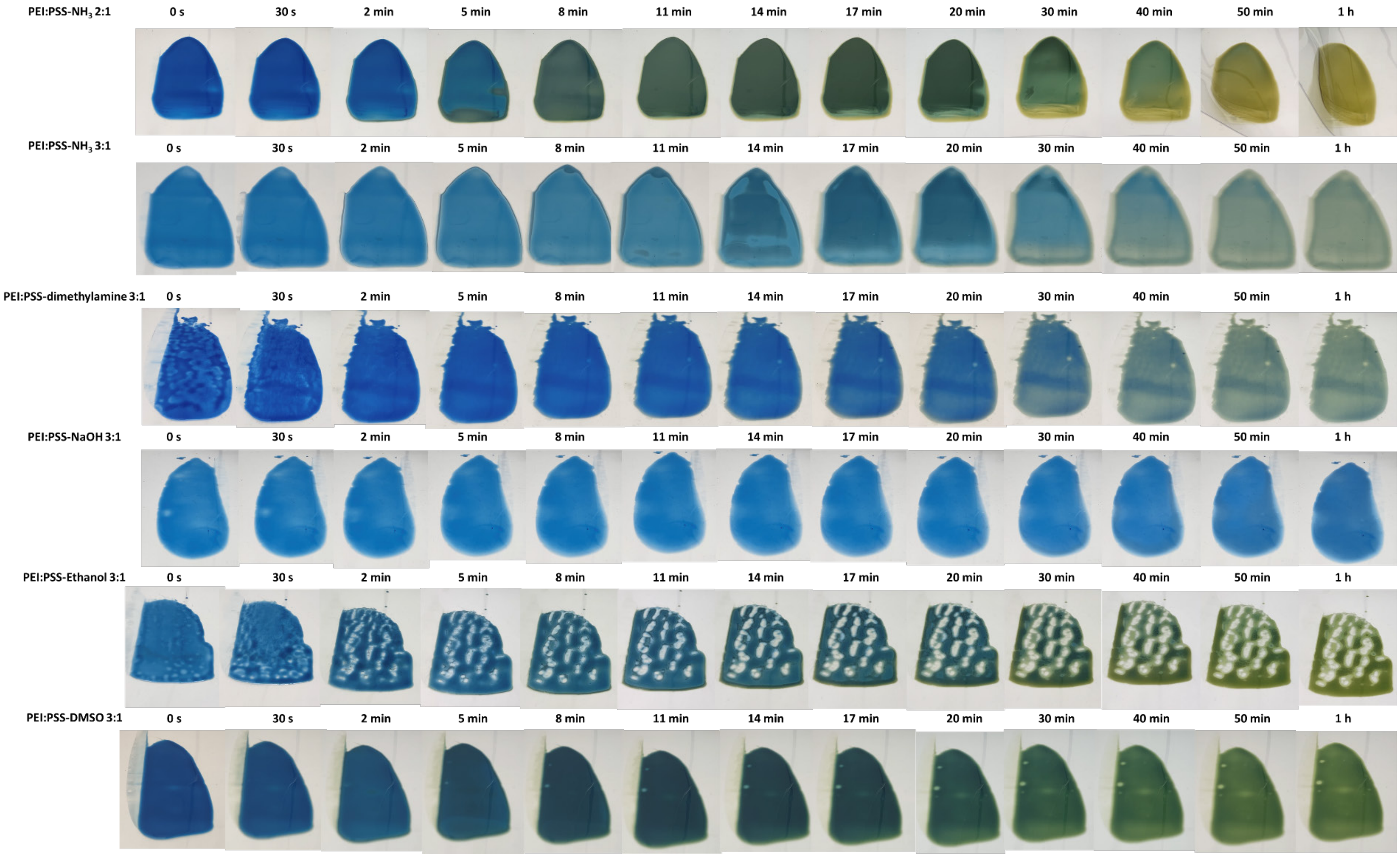

Figure S8. Screenshot pictures of different films with thymol blue drying vs time. Same amount of thymol blue was added into the solutions (~ 0.001 g in 9 g solution).

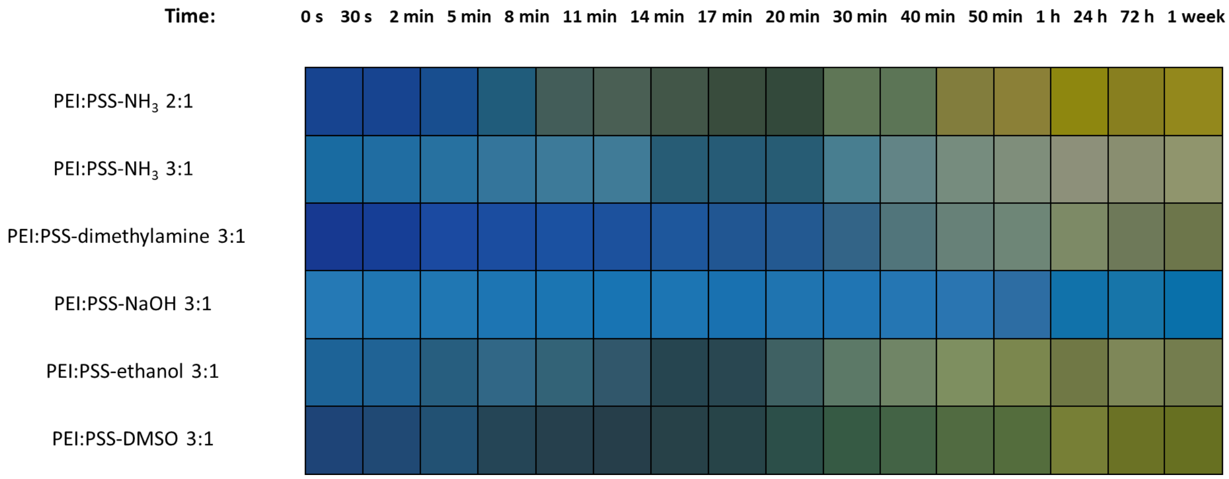

Figure S9. Color change vs time of different samples. The color of the same point was tracked vs time. For samples with NH_3, the color became darker in the middle region which matches with the turbidity increase.

Similarly, CMYK (cyan, magenta, yellow, key (black)) values can be obtained from the colors. Here, we focused on the Y/C ratio which determines how green the color is. From Figure S1c, we can obtain roughly the ratio trend for pH 7-13 as a reference (Figure S10). As shown in Figure S1a, the yellow color from PSS itself became stronger with the pH increase. In principle, as pH increases, a more blue color should be obtained. However, due to this increase of yellow, an increase in Y/C was observed from pH 9 to 12. Thus instead of one equation, the Y/C ratio was first calculated for each point and from the value, it was assigned to a region then a certain equation was used for calculation. For example, if Y/C value = 0.5, it can be either pH 9-10 or pH 11-12. According to previous points, it already reached pH 10, so this point should fall into the region pH 9-10 and the pH would be calculated based on equation 3.

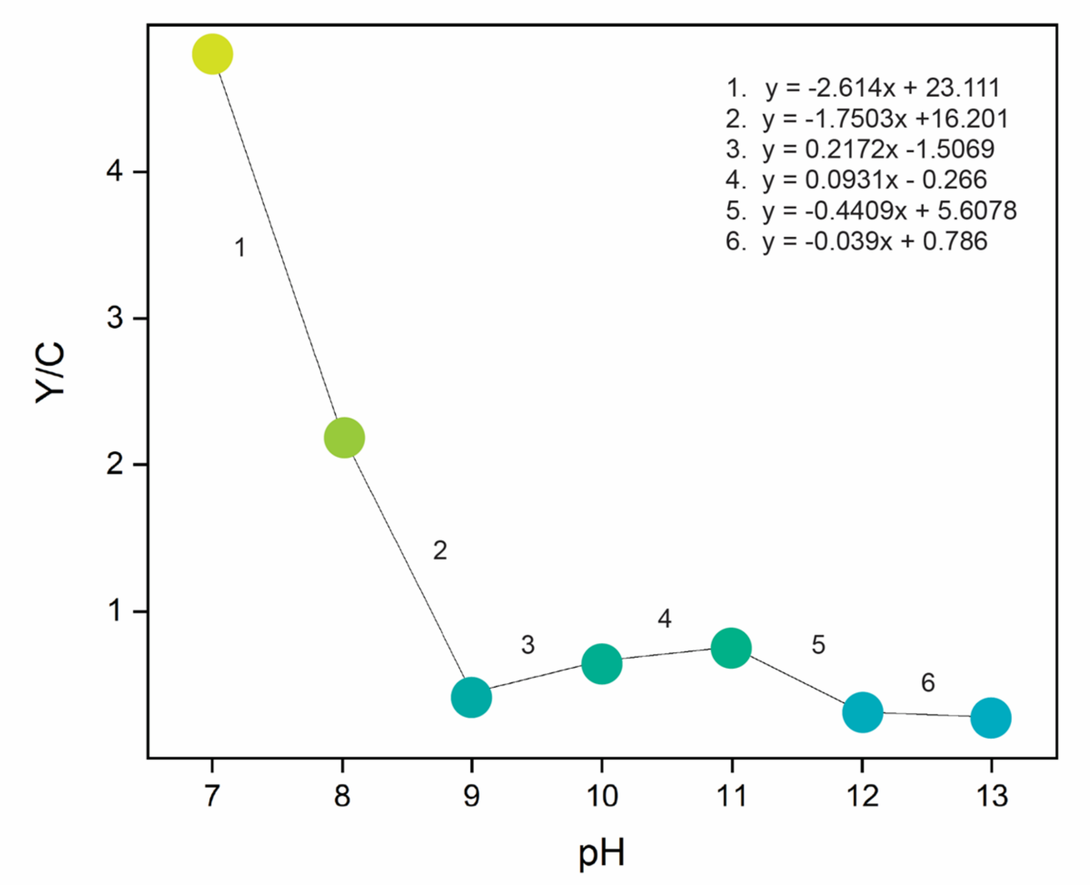

Figure S10. Y/C value vs pH obtained from the reference color system. According to the Y/C value of each point, it can be assigned into a pH region then the pH can be calculated using the right equation.

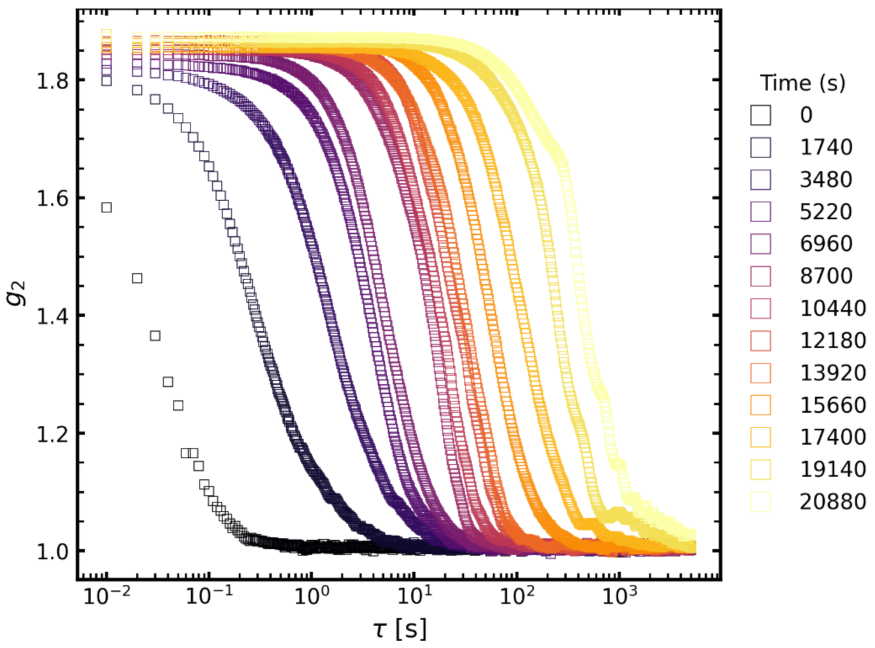

Figure S11. Example g_2 intensity autocorrelation functions for the dimethylamine sample shown in Figure 4.

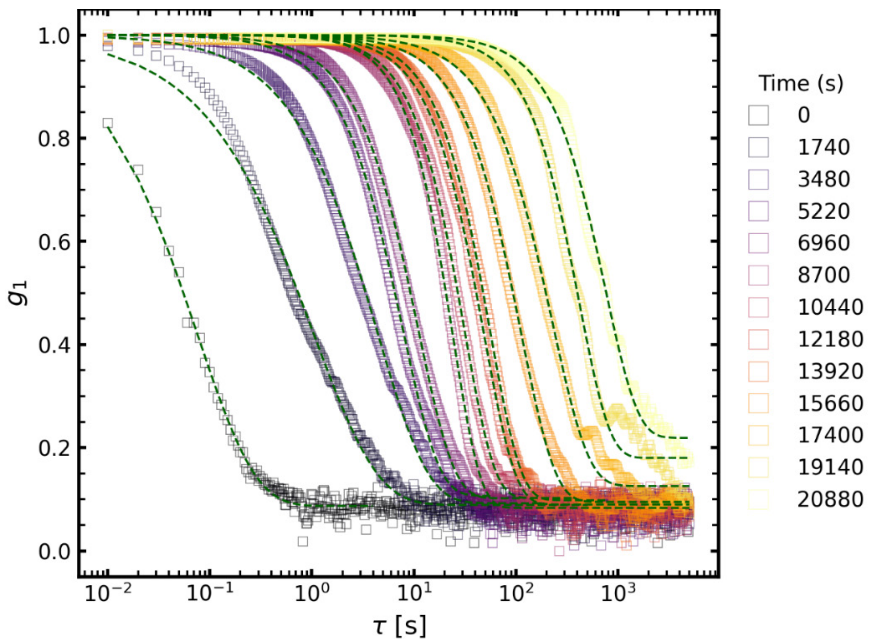

Figure S12. Example g_1 normalized field correlation functions for the dimethylamine sample shown in Figure 4.

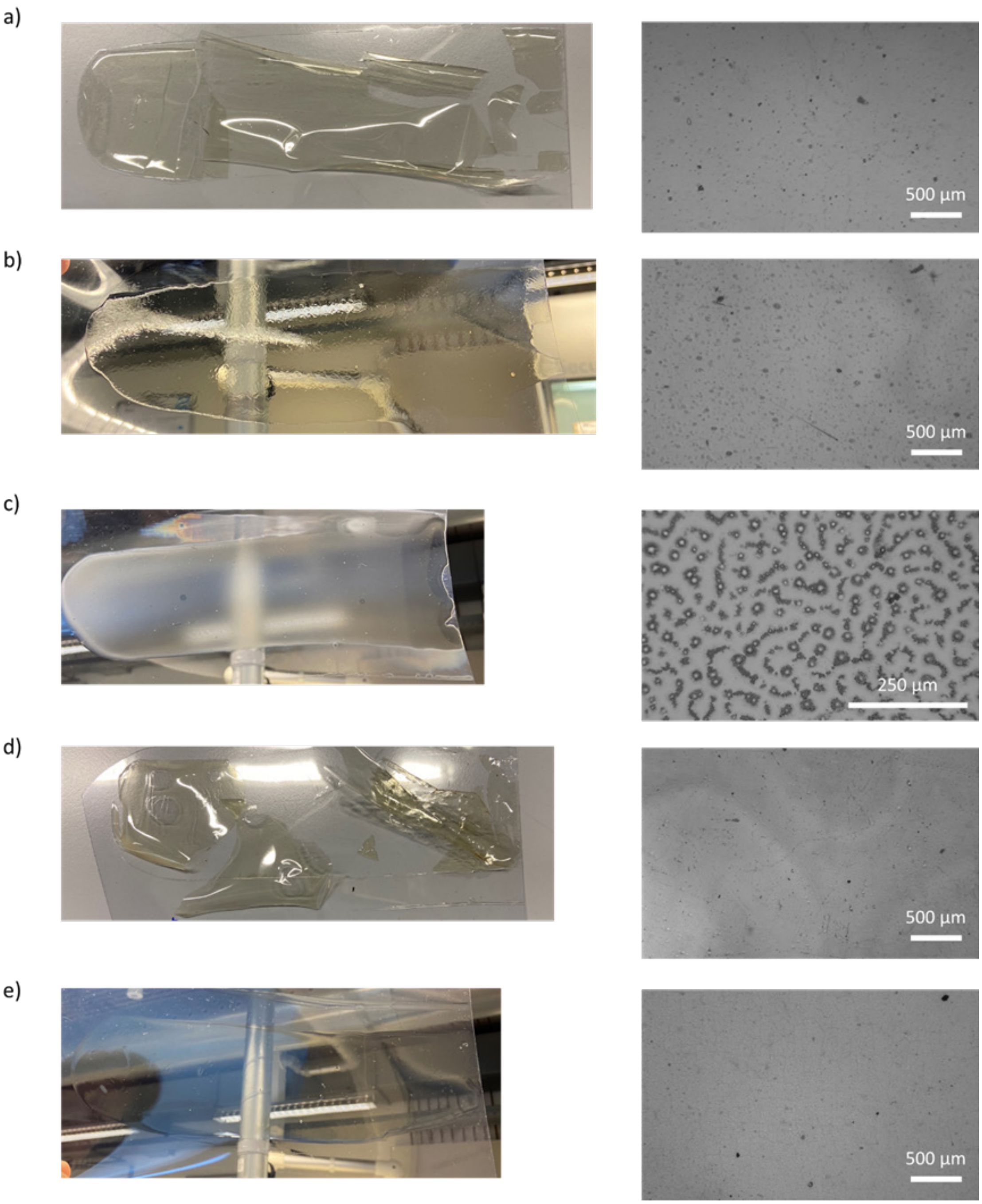

Figure S13. Physical appearance of dry PEI/PSS films with a) PSS-NH_3, b) PSS-dimethylamine, c) PSS-NaOH, d) PSS-ethanol, and e) PSS-DMSO at a ratio of 2:1. Images were taken 24 h after casting.

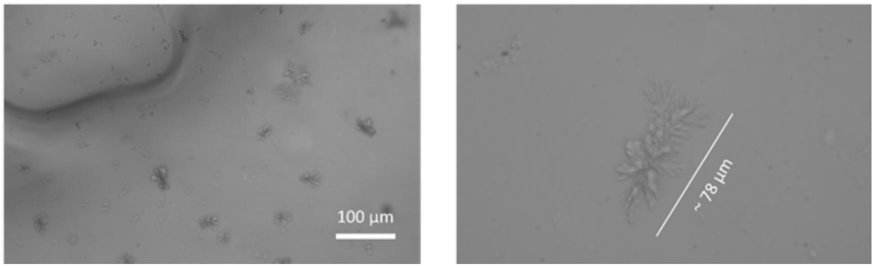

Figure S14. PSS-DMSO sample showed spherulite-like crystals. Images were taken 24 h after casting.

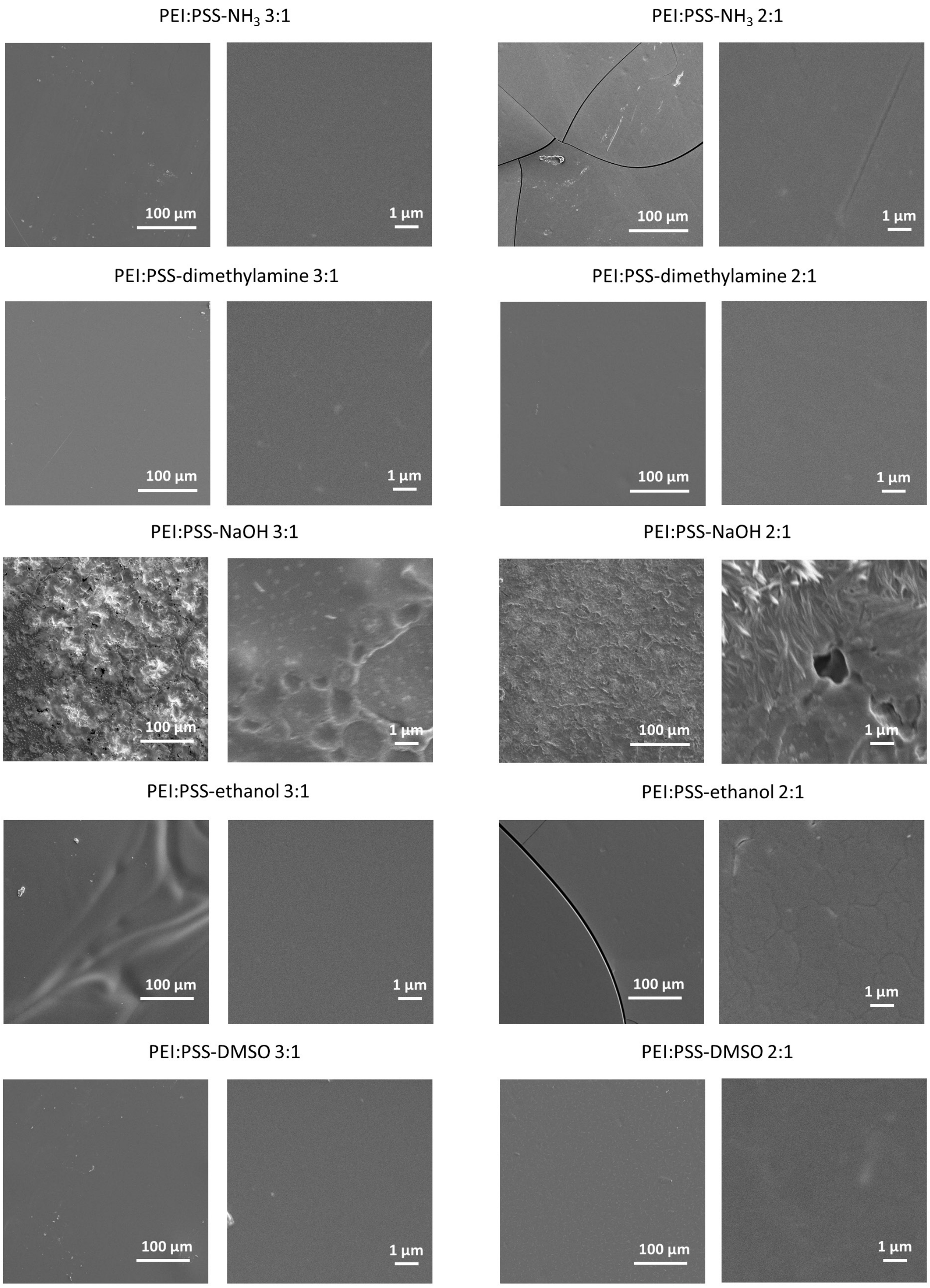

Figure S15. SEM images of different samples. Due to vacuum, cracks could be generated for PEI:PSS at a ratio of 2:1 samples.

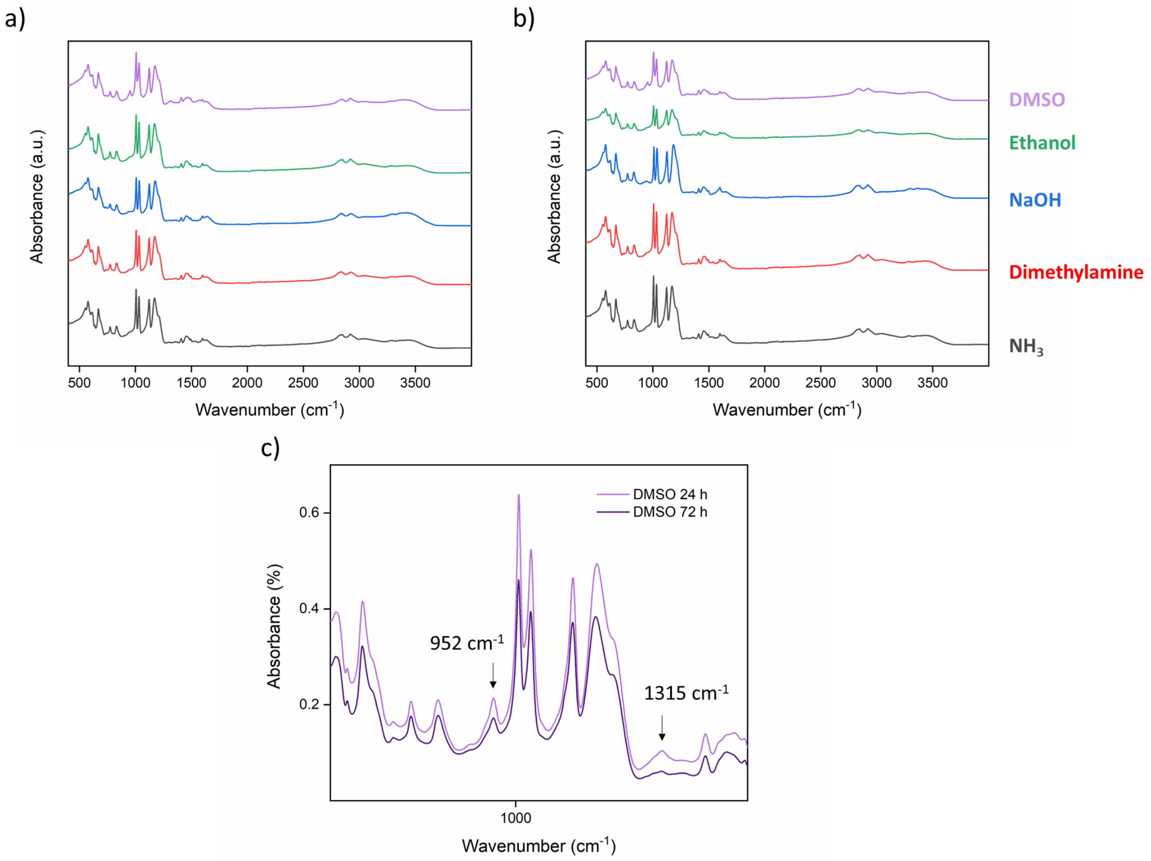

Figure S16. IR spectra of PEI:PSS samples at a ratio of 3:1: a) 24 h, b) 72 h, and c) PEI/PSS-DMSO vs time where DMSO peaks decreased.

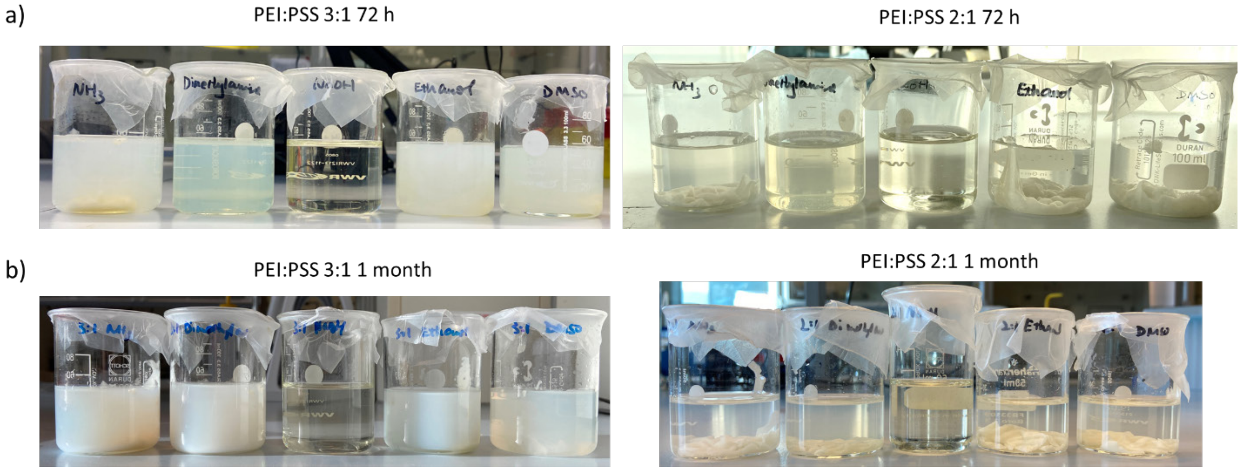

Figure S17. Swelling tests of films (from left to right: -NH_3, -dimethylamine, -NaOH, -ethanol, -DMSO) a) 72 h and b) 1 month after casting.

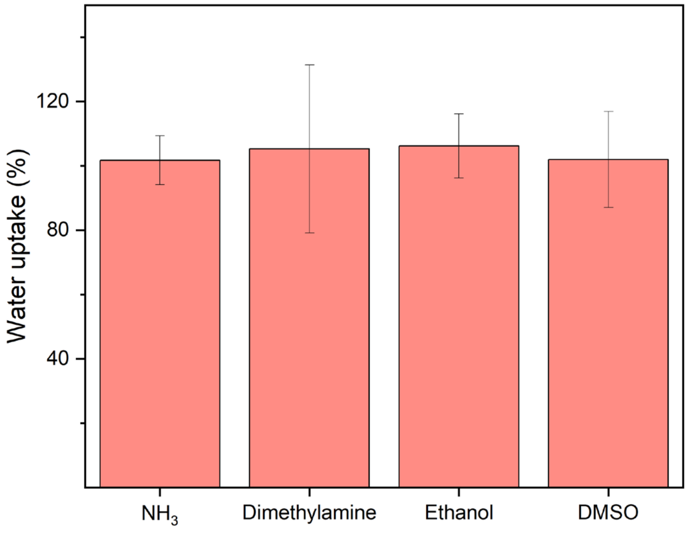

Figure S18. Water uptake of PEI:PSS samples at a ratio of 2:1.

Table S1. The thickness (μm) of tensile samples vs time (* Cracked samples that could not be measured).

Time / Ratio		24 h	72 h	1 month
PEI:PSS 3:1	NH_3	97 ± 4	90 ± 5	
	Dimethylamine	98 ± 6	85 ± 7	94 ± 4
	NaOH	118 ± 2	110 ± 5	
	Ethanol	108 ± 8	104 ± 15	
	DMSO	125 ± 11	109 ± 11	122 ± 3
PEI:PSS 2:1	NH_3	77 ± 6*		
	Dimethylamine	104 ± 11	71 ± 2*	
	NaOH	119 ± 7	103 ± 7	
	Ethanol	73 ± 25*		
	DMSO	106 ± 10	105 ± 11	108 ± 5*

Table S2. Tensile measurements (Young's modulus/Tensile strength/Elongation) of PEI:PSS at a ratio of 2:1 samples vs time (* Cracked samples that could not be measured).

Type	24 h	72 h	1 month
NH_3	*		
Dimethylamine	2436.7 ± 160.1/6.24 ± 3.78/0.41 ± 0.33	*	
NaOH	1693.1 ± 111.8/15.27 ± 2.64/3.53 ± 1.64	1539.6 ± 153.5/11.08 ± 0.86/3.30 ± 0.48	
Ethanol	*		
DMSO	623.0 ± 217.7/1.42 ± 0.41/0.94 ± 0.85	1296.4 ± 269.0/2.39 ± 0.30/0.38 ± 0.13	*

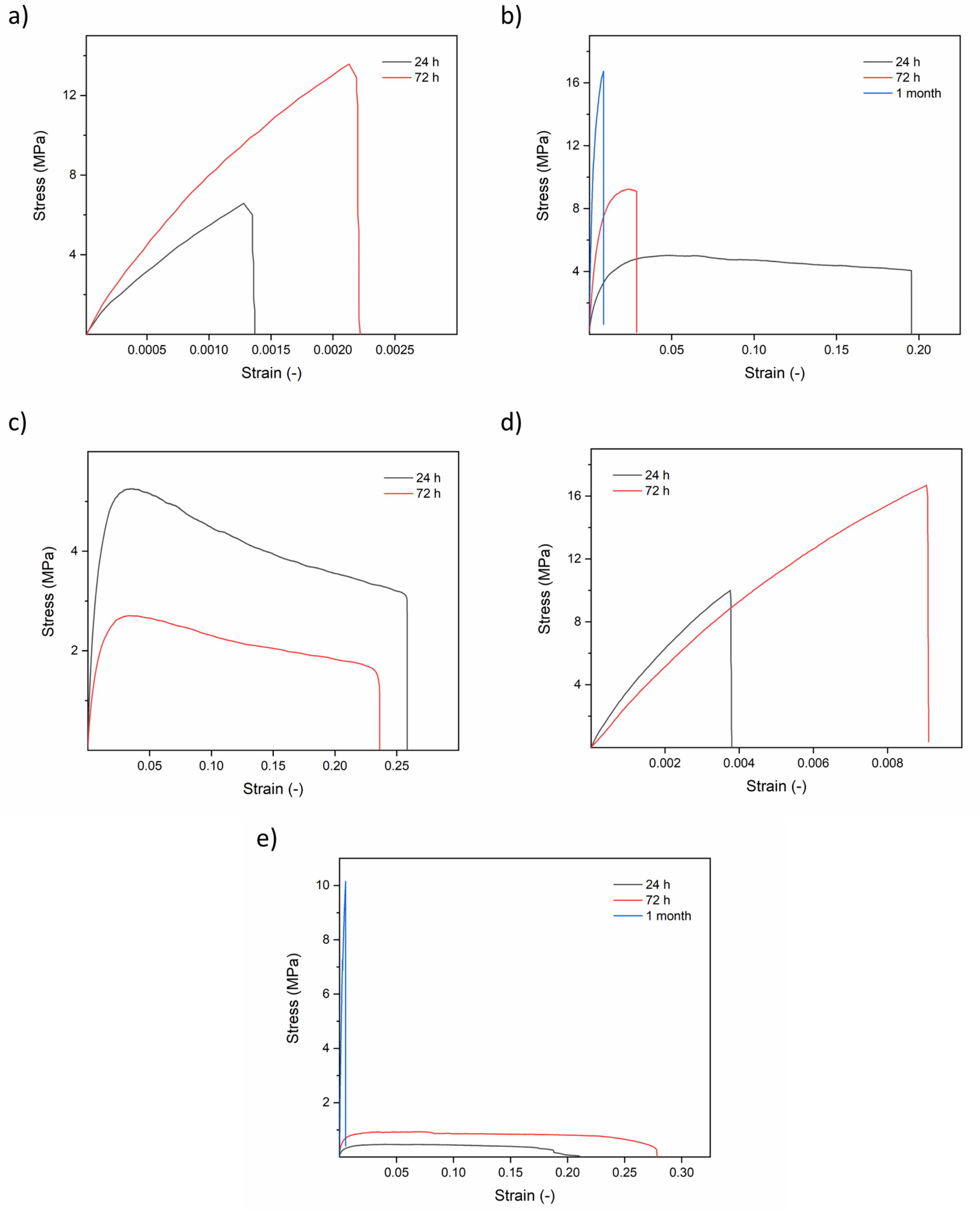

Figure S19. Representative stress-strain curves of PEI:PSS samples at a ratio of 3:1 with: a) PSS-NH_3, b) PSS-dimethylamine, c) PSS-NaOH, d) PSS-ethanol, and e) PSS-DMSO vs time.

Appendix 1. Calculation of initial molar evaporation of volatile base

To calculate the molar evaporation of volatile base, the difference between the evaporation rate of water and volatile base just after casting and after 1000 s assessed. To approximate this, the slopes of the mass profile, corrected for the initial mass of the applied coating film, were determined. This correction is important, since the applied coating mass varied slightly between samples, yet gives an indication of the total surface area of the coating film (since the thickness of the film is constant due to the application *via* casting rod, assuming similar density), which is an important factor for evaporation rates. To visualize this, the initial corrected mass decrease is shown in Figure S20, together with the determined slopes.

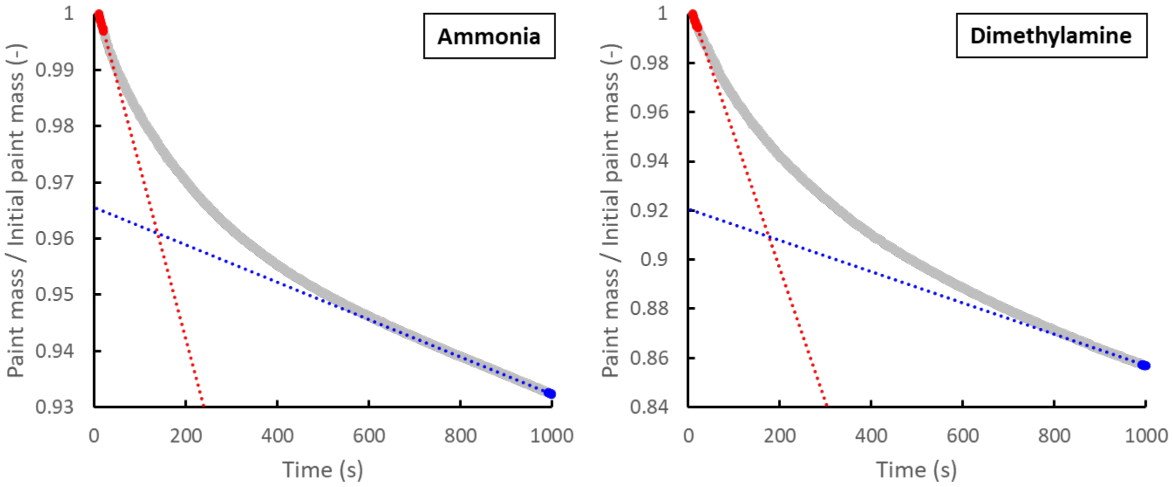

Figure S20. Normalized mass decreases over time for ammonia and dimethylamine samples. Indicated are the slopes at t = 10-20 s and t = 990-1000 s.

The obtained decreases in normalized mass over time were used as follows:

$$Approx. evaporated\ base\ [mol\ s^{-1} g^{-1}] = -\frac{slope_{t=10\rightarrow 20\ s}[s^{-1}] - slope_{t=990\rightarrow 1000\ s}[s^{-1}]}{MW_{volatile\ base}\ [g\ mol^{-1}]}$$

Here, the evaporation of water is assumed equal between the two different time ranges. Furthermore, we take the times from t = 10-20 s as initial values, to remove distortions caused by vibrations of the balance right after placing the sample. The values obtained from this calculation are respectively:

$$Approx.evaporated\ ammonia = -\frac{-3.06 * 10^{-4} - (-3.37 * 10^{-5})}{17.031}$$

$$= \mathbf{1.60 * 10^{-5}\ mol\ s^{-1}\ g^{-1}}$$

$$Approx.evaporated\ dimethylamine = -\frac{-5.44 * 10^{-4} - (-6.93 * 10^{-5})}{45.085}$$

$$= \mathbf{1.05 * 10^{-5}\ mol\ s^{-1}\ g^{-1}}$$

The higher value for ammonia indicates a higher molar rate of evaporation per gram of applied coating than for dimethylamine. This supports our hypothesis that ammonia evaporates faster, and pH decreases more rapidly in these samples.

Notably, both mass curves also converge to a roughly linear decrease, as observed in the original, longer timescale plots. This seems to indicate that most of the volatile base evaporates initially, and the water evaporation is the main contributor after a certain time. This is further supported by the fact that after 1000 s, the mass decrease is roughly the same as the initial added weight of volatile base. We do stress that the last portion of volatile base that evaporates might still significantly change the pH, even though it is not as clearly observable in a mass decrease. To visualize this further, we add the following Figure S21, where it is visible the weight decrease is roughly the same as initially added volatile base (6.5 wt% and 17.3 wt% added for ammonia and dimethylamine respectively):

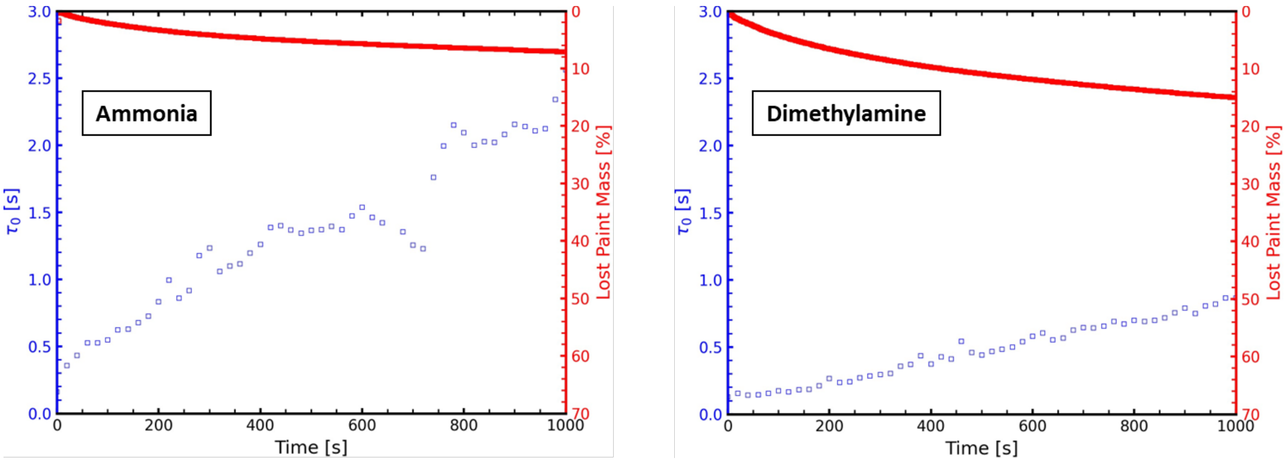

Figure S21. Lost paint mass and τ_0 over time during initial drying stages. Note that the lost paint mass at t = 1000 s is roughly equal to the amount of added ammonia (6.5 wt%) and dimethylamine (17.3 wt%).
